# CardioMetaHybridOptimizer as a behaviorally adaptive multi-phase metaheuristic framework for interpretable cardiovascular disease diagnosis

**DOI:** 10.1186/s12859-026-06489-x

**Published:** 2026-06-09

**Authors:** Ahmed Kateb Jumaah Al-Nussairi, Yasser Taha Alzubaidi, Ali k. Abdul Raheem, Saleem Malik, Kabul Khudaybergenov, Ahmed Shakir Al-Hiti, Quadri Noorulhasan Naveed, Shafat khan, Aseel Smerat, Mequanent Erkie Ayele

**Affiliations:** 1 Dean of the Technical Engineering College, University of Manara, Maysan, Iraq; 2 Mathematics Department, College of Basic Education, University of Misan, Misan, 62001 Iraq; 3 Department of Medical Instrumentation Engineering Techniques, Al-Safwa University College, Karbala, Iraq; 4 University of Warith Al-Anbiyaa, Karbala, Iraq; 5 CSE Department, P A College of Engineering, Mangalore, 574153 India; 6 Department of Applied Informatics, Kimyo International University in Tashkent, Tashkent, Uzbekistan; 7 Dept. of Medical Instrument Tech. Engineering, Faculty of Engineering Techniques, University of Almaarif, Ramadi, 31001 Iraq; 8 Dept. of Electrical Engineering, Faculty of Engineering, University of Anbar, Ramadi, 31001, Iraq; 9 Department of Computer Science, College of Computer Science, King Khalid University, Abha, Kingdom of Saudi Arabia; 10 Department of Biosciences, Saveetha School of Engineering, Saveetha Institute of Medical and Technical Sciences, 602105, Chennai, India; 11 Hourani Center for Applied Scientific Research, Al-Ahliyya Amman University, Amman, 19328 Jordan; 12 Department of Electrical and Computer Engineering, Gafat Institute of Technology, Debre Tabor University, Debre Tabor, Ethiopia

**Keywords:** Cardiovascular disease prediction, Optimization technique, Dynamic mutation

## Abstract

Cardiovascular disease prediction is delayed by high-dimensional clinical data and heteroginity. There is a need for decision-support system that can select relevant features. We propose a Cardio Meta Hybrid Optimizer (CMHO) framework designed to enhance feature selection and predictive accuracy in cardiac risk assessment.The CMHO framework integrates three metaheuristic algorithms—Lion Optimization (LO), Marine Predators Algorithm (MPA), and Manta Ray Foraging Optimization (MRFO)—enhanced with adaptive switching, dynamic mutation, and iterative local search (ILS). The framework was evaluated on five benchmark datasets: Cleveland, Hungarian, Statlog, Switzerland, and Long Beach VA. We uesd a CNN-LSTM architecture for classification, validated through stratified tenfold cross-validation with 10 independent repetitions. Performance was benchmarked against RFE, GA, PSO, GWO, and Lasso using ANOVA to confirm statistical significance. The CMHO-integrated CNN-LSTM model achieved a accuracy of 96.1%, outperforming traditional feature selection methods by 3%–5% (*p* < 0.05). The framework demonstrated stability and clinical interpretability by selecting validated biomarkers—including thalassemia, chest pain type, and maximum heart rate—with a Stability Selection Index (SSI) > 0.90.The CMHO framework provides a robust and interpretable tool for cardiovascular risk assessment. By navigating high-dimensional data across diverse populations, it offers a reliable computational approach for clinical decision support in cardiology.

## Introduction

The rapid digitization of healthcare systems has led to the generation of large-scale, high-dimensional clinical datasets, offering significant opportunities for early detection of cardiovascular disease (CVD). However, these datasets often contain redundant, noisy, and irrelevant features, which introduce the *curse of dimensionality*. This affects machine learning models by increasing computational complexity, reducing generalization ability, and leading to overfitting. In clinical settings, such limitations can impact diagnostic reliability and delay intervention, making efficient and interpretable feature selection a critical requirement.

Feature selection techniques have been adopted to address these challenges by reducing dimensionality and improving model performance. Nevertheless, existing approaches—including traditional statistical methods and metaheuristic-based algorithms—face significant limitations. Many methods struggle to maintain an effective balance between global exploration and local exploitation, often resulting in premature convergence and suboptimal feature subsets. Furthermore, several studies lack robustness across heterogeneous datasets and fail to identify clinically meaningful features, limiting their application in real-world healthcare scenarios.

These limitations highlight a important research gap: the absence of a robust, adaptive, and interpretable feature selection framework that can balance exploration and exploitation while maintaining consistent performance across diverse cardiovascular dataset**s**. Addressing this gap is essential for developing reliable predictive models that can support clinical decision-making.

To overcome these challenges, this study proposes CardioMetaHybridOptimizer (CMHO), a novel multiphase hybrid optimization framework for feature selection. The proposed method integrates the complementary strengths of the Lemur Optimizer for global exploration, the Marine Predators Algorithm for adaptive refinement, and Manta Ray Foraging Optimization for local exploitation. In addition, the framework incorporates Iterative Local Search (ILS) and a dynamic mutation mechanism to prevent stagnation and enhance search diversity. This structured hybridization enables a more effective traversal of the feature space, leading to the selection of compact, stable and clinically relevant feature subsets.

The effectiveness of the proposed approach is evaluated on five benchmark cardiovascular datasets (Cleveland, Hungarian, Statlog, Switzerland, and Long Beach VA) using multiple classifiers. A rigorous experimental protocol with stratified cross-validation and repeated runs is employed to ensure robustness and avoid data leakage. Furthermore, statistical validation and feature stability analysis are conducted to assess both performance significance and interpretability.

The main contributions of this work are as follows:

- Development of a multiphase hybrid optimizer: We introduce CardioMeta HybridOptimizer, a three-stage framework that solves the exploration–exploitation imbalance. Unlike single-algorithm approaches, it integrates the Lemur Optimizer for global search, the Marine Predators Algorithm for adaptive refinement, and Manta Ray Foraging Optimization for precise exploitation.
- Enhanced convergence and stability: By incorporating Iterative Local Search (ILS) and a dynamic mutation mechanism, the proposed framework overcomes the common research gap of “premature convergence” and “local optima entrapment” found in conventional metaheuristics.
- Optimization for high-dimensional clinical data: The framework is tuned to handle the high dimensionality and noise characteristic of cardiovascular datasets, ensuring that the selected feature subsets are significant but also clinically interpretable for diagnostic decision-making.
- Empirical validation and performance superiority: Through extensive experiments on real-world heart disease datasets, we demonstrate that the proposed hybrid approach outperforms state-of-the-art algorithms in terms of classification accuracy, subset reduction rate, and computational efficiency**.**

## Preliminaries

Cardiovascular disease prediction feature selection is fast and reliable with a hybrid architecture combining three bio-inspired algorithms. Each method has been used to optimization tasks, but CMHO’s structural integration with dynamic control mechanisms permits adaptive exploration, refinement, and exploitation stages.

### Lemur optimizer

The Lemur Optimizer (LO) replicates leaping and exploring [1]. For global exploration, CMHO generates a diverse starting population of feature subsets using LO. Stochastic search gives substantial solution space coverage for high-dimensional cardiovascular datasets. The innovation is how LO’s population progresses beyond experimental output.

### Marine predators algorithm

MPAs simulate marine predators’ synchronized hunting and adaptive foraging [2]. CMHO focuses on LO-identified high-potential zones using MPA as an intermediate refining step to balance exploration and exploitation. Unlike most programs, MPA refines candidate solutions in context using real-time fitness updates from prior phases.

### Manta ray foraging optimization

Manta Ray Foraging Optimization (MRFO) mimics manta ray somersault foraging to find optimal places [3]. Early-developed high-quality feature subsets are refined in CMHO’s concentrated exploitation phase, MRFO. Through dynamic mutation and recurrent local search, CMHO can avoid local optima and perform multi-phase optimization instead of algorithmic execution.

### Hybrid metaheuristics

CMHO innovates beyond merging LO, MPA, and MRFO [4]. Real-time fitness evaluation-based adaptive phase-switching, dynamic mutation for variety, and iterative local search (ILS) for convergence are used instead. Using structural hybridization, CMHO can improve feature selection, convergence, and prediction on high-dimensional cardiovascular datasets by combining algorithms’ strengths and shortcomings. CMHO purposely combines these algorithms into a behaviorally adaptive framework for remarkable synergy [5]. Simply concatenating cannot do this.

## Problem statement

Healthcare relies on CVD prediction for early diagnosis, customized treatment, and better patient outcomes [6]. This issue uses high-dimensional clinical, demographic, and lifestyle medical datasets. The objective is to choose the most important factors and build reliable predictive models to classify patients into high- or low-risk cardiovascular risk groups [7]. Our goal is twofold:

*Feature selection***:** Identify a compact subset of input variables that preserves maximum predictive power while discarding irrelevant or redundant data.

*Classification***:** Train a model that, given the selected features, assigns each patient to one of *m* predefined risk categories.

Mathematically, we denote the dataset as $X=\left\{{x}_{1}, {x}_{2},\dots , {x}_{n}\right\}$, where each ${x}_{i}\in {R}^{d}$ is the *d-dimensional* feature vector for patient *i*. The true label vector is $y={\left\{{y}_{1}, {y}_{2},\dots , {y}_{n}\right\}}^{T}$, where each ${y}_{i}$ $\in C=\left\{{C}_{1},{C}_{2},\dots ,{C}_{m}\right\}$ indicates the cardiovascular risk category of patient *i*.

We define a feature-index set *F* ⊂$\left\{\mathrm{1,2},\dots ,d\right\},$ and denoted by ${X}_{F}$ $=\left\{{x}_{i}^{F} \right|i=\mathrm{1,2},\dots n$} the projection of *X* onto the coordinates in *F*.

A classifier is a function $f: {R}^{\left|F\right|}\to C$ that maps each reduced feature vector ${x}_{i}^{F}$ to a predicted label$\widehat{{y}_{i}}=\mathrm{f} ({x}_{i}^{F}$), and we collect all predictions in the vector $\widehat{y}= {\left\{{\widehat{y}}_{1},{\widehat{y}}_{2},\dots , {\widehat{y}}_{n}\right\}}^{T}$

To quantify misclassification, we use a loss function$L({\widehat{y}}_{i} , {y}_{i})$, with the zero‑one loss defined by $L({\widehat{y}}_{i} , {y}_{i})$= 1(${\widehat{y}}_{i} \ne {y}_{i}$), where 1{⋅}is the indicator function. The empirical risk (average loss) over the dataset is$L \left(f :F\right)=\frac{1}{n}\sum_{i=1}^{n}L\left(f\left({x}_{i}^{F} \right),{y}_{i}\right)$, which under zero‑one loss simplifies to the classification error *E (F)* =$\frac{1}{n}\sum_{i=1}^{n}1\left(f\left({x}_{i}^{F} \right)\ne {y}_{i}\right)$.

Our goal in the CardioMetaHybridOptimizer framework is to select the feature subset *F* and classifier *f* from a hypothesis space *H* so as to minimize this empirical risk:

$$F\subset {\{1, 2, \dots d\}}_{f \epsilon H}^{min}=\frac{1}{n}\sum_{i=1}^{n}L\left(f\left({x}_{i}^{F} \right),{y}_{i}\right).$$

Here, *H* denotes the set of all candidate classifiers (e.g., SVMs, decision trees, or neural networks parameterized by θ) from which our algorithm selects the model that best balances accuracy and complexity.

Let $F\subseteq \{1,\dots ,d\}$ be the selected feature set of size $\mid F\mid$, and let Acc *(F)* denote the classification accuracy on a held‐out validation set. We then maximize $J\left(F\right)=\alpha Acc\left(F\right)-\beta \mid F\mid /d$, subject to $\alpha +\beta =1$, $\alpha ,\beta \ge 0$. Here the term $\mid F\mid /d$ is the fraction of features chosen (so that smaller subsets incur a smaller penalty), and α, β trade off accuracy versus subset compactness. In all experiments we set α = 0.7 and β = 0.3 after a brief grid search, thereby prioritizing predictive performance while still discouraging large feature sets.

Alternatively, one can view our framework as solving the bi‐objective problem

$$F\subseteq min \{1,\dots ,d\} \{E(F),\mid F\mid /d\},$$

where *E(F)=1-Acc(F)*.

Unlike GA + PSO and DE + SA, which combine algorithms sequentially, CMHO dynamically balances exploration and exploitation using real-time fitness feedback. The structured multi-phase design—Lemur Optimizer for diverse global exploration, Marine Predators Algorithm for intermediate refinement, and Manta Ray Foraging Optimization for focused exploitation—guides candidate solutions to high-quality feature subsets while maintaining population diversity. Dynamic mutation, fitness-ranked adaptive scaling, and iterative local search (ILS) help the algorithm avoid local optima and stagnation, which classic hybrids often struggle with. CMHO converges quicker and more reliably on high-dimensional cardiovascular datasets due to its adaptive coordination, giving it a theoretical and practical edge over hybrid systems without context-aware phase-switching algorithms.

## Literature survey

In predicting cardiovascular disease, good feature selection and classification approaches are vital for boosting model performance, lowering computing overhead, and promoting interpretability [8, 9]. Many scholars have developed algorithms and frameworks to overcome these issues [10–12]. Table 1 summarizes popular hybrid research works.

**Table 1:** Related works

References	Approach type	Pros	Cons
[42]	SVM, Bayes Net, Functional Tree with Best-First FS	Uses feature selection; multiple ML models compared	Limited dataset (Cleveland); small feature size
[43]	Naïve Bayes Classification	Simple, fast, high interpretability	Poor for large dataset and nonlinear data
[44]	Naïve Bayes, Bagging, J48	Ensemble outperforms single model	Not optimized feature selection
[45]	ANN + LVQ	Uses ANN for medical variables	Low accuracy for clinical use
[46]	Hybrid MLP + SVM	Combines two classifiers	No feature selection optimization
[47]	XGBoost + SMOTE + SHAP (Explainable AI)	High accuracy with optimized features; provides model interpretability (Explainable AI); uses combined public/private datasets	May require more computational resources for optimization and explanation generation
[48]	SVM + Jellyfish Optimization Algorithm (Feature Selection)	High convergence speed and robust feature selection; resistant to outliers; uses comprehensive Cleveland data set	Potential for increased complexity in implementing metaheuristic algorithms
[7]	ML-HDPM (MLDCNN + AEHOM + UCOM)	Addresses data imbalance and feature selection well; uses multi-layer deep learning	Deep learning models can be computational intensive and complex to tune
[49]	CNN for Tabular Data + SHAP (Explainable AI)	High specificity (100%) and interpretability; adapts CNNs for structured clinical data	High specificity may come at the cost of sensitivity in some cases; novel approach for tabular data
[50]	Hybrid BLSTM + BGRU (Deep Learning)	Excellent performance with sequence data (ECG signals); captures long-range dependencies	Requires specialized data (ECG signals) rather than standard clinical features

Filter Methods rate features without classifier performance using statistical metrics as ReliefF, Chi-Square, and Mutual Information [13]. Studies [14–16] showed that enhanced classification accuracy in cardiovascular datasets. These approaches are easy to use, classifier-independent, and computational cheap, but they ignore feature interdependencies and struggle with high-dimensional datasets, resulting to inferior feature subsets in complicated scenarios [17].

GA and PSO wrapper methods optimise feature subsets for a classifier [18]. Classifier feedback improves performance and problem-specific optimization [19, 20]. But they’re computational intensive, prone to premature convergence, and unsuitable for large datasets. Hybrid techniques balance computational efficiency and selection quality by combining filter and wrapper strengths [18]. [21] provided a more realistic hybrid architecture than solo methods. These approaches combine exploration and exploitation well, but they are difficult to implement and require extensive parameter adjusting [22, 23].

Ant Colony Optimisation (ACO) [24] and Grey Wolf Optimisation (GWO) [25, 26] are exploratory and adaptable metaheuristic algorithms. They need further fine-tuning due to local optima. Adaptive approaches include Manta Ray Foraging Optimisation (MRFO) [27–29] and Marine Predator Algorithms (MPA) [30, 31] increase convergence and balance exploration and exploitation. They can’t avoid local optima and lack real-world tests. CNNs and LSTMs [32] can extract features from high-dimensional raw data and capture complex, non-linear patterns. Despite these advantages, their processing needs and overfitting risk limit their use in resource-constrained settings [32–38].

Differential Evolution with Simulated Annealing [39] and PSO with dynamic mutation [40] are hybrid metaheuristic frameworks that improve performance by combining metaheuristic algorithms with complementary methods. Some of these frameworks improve convergence and search space variety, but they are complicated and take longer to design and tune. Due to their performance and interpretability, SVMs and Random Forests are still used for structured datasets [41] However, effective feature selection and raw or unstructured data issues could limit performance without sufficient preparation.

Many hybrid metaheuristics with dynamic mutation have been proposed, but most of them have two major drawbacks: (i) they use pairwise combinations of algorithms (e.g., GA + PSO, DE + SA, WOA + GWO), which often lack a structured division of exploration–transition–exploitation phases, resulting in premature convergence; and (ii) their dynamic mutation strategies operate across all population members, ignoring data The suggested CMHO addresses this gap by using a tri-phase hybrid architecture (LO → MPA → MRFO) to assign each algorithm a function based on its strengths. CMHO also uses a selective dynamic mutation and ILS method to adjust the mutation rate based on feature-selection difficulty and stagnation detection, making subset finding more stable. This structured hybridization and adaptive mutation strategy distinguishes CMHO from previous methods and addresses high dimensionality, redundant characteristics, and medical interpretable selection, which are understudied.

## Methods and materials

**Fig. 1:**
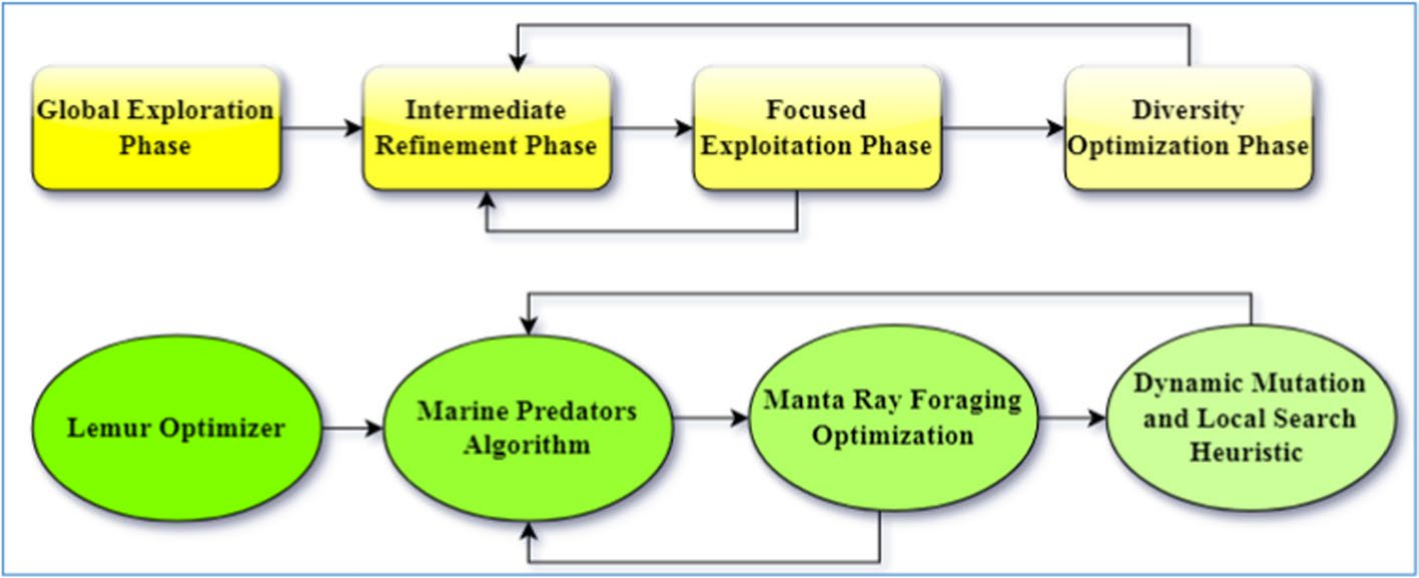
Conceptual view of CMHO

Figure 1 shows how the CardioMetaHybridOptimizer balances exploration, refinement, and exploitation through a systematic succession of optimization steps to cover search space and improve convergence efficiency. The Lemur Optimizer undertakes population-based exploration in the Global Exploration Phase to minimize premature convergence by diversifying viable solutions. The Intermediate Refinement Phase begins if fitness increases. Diversity mechanisms may reinitialize the population with more exploration if no improvement occurs.

The Intermediate Refinement Phase uses the Marine Predators Algorithm to refine candidate solutions while balancing exploration and exploitation. Following fitness enhancement, Manta Ray Foraging optimization refines high-quality solutions through extensive local search in the Focused Exploitation Phase.

If no progress is made, the flow might return to the Intermediate Refinement Phase. The Diversity optimization Phase also uses dynamic mutation and local iterative search to avoid local optima entrapment. This phase adds solution variety and can return to the Intermediate Refinement Phase to stabilize the search The CMHO keeps optimizing until it reaches the max number of iterations or sees no progress after several cycles. CMHO is useful for difficult classification tasks because to its organized flow, which enables variety and exploitation.

**Algorithm 1:**
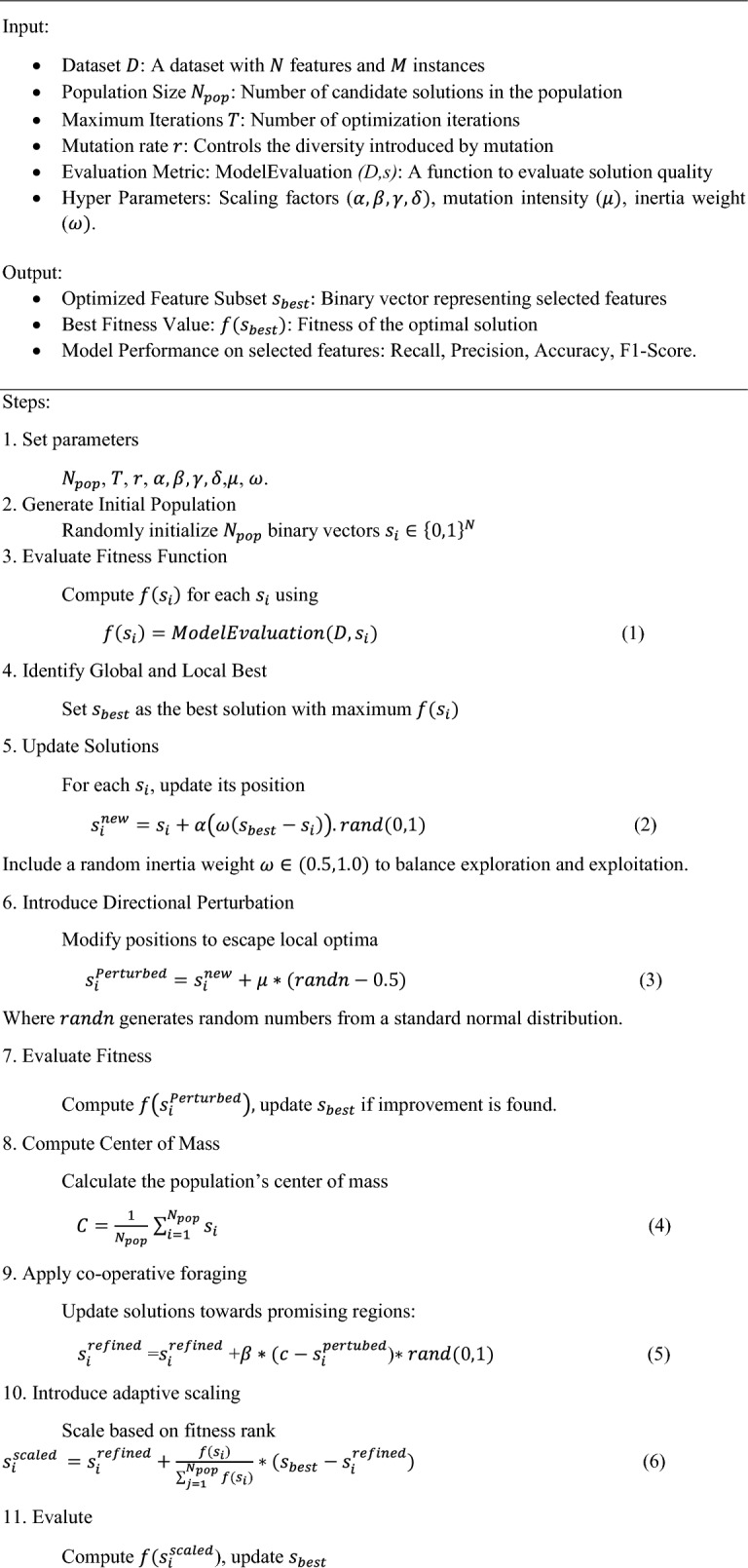
CardioMetaHybridOptimizer (CMHO) Algorithm

**Figure:**
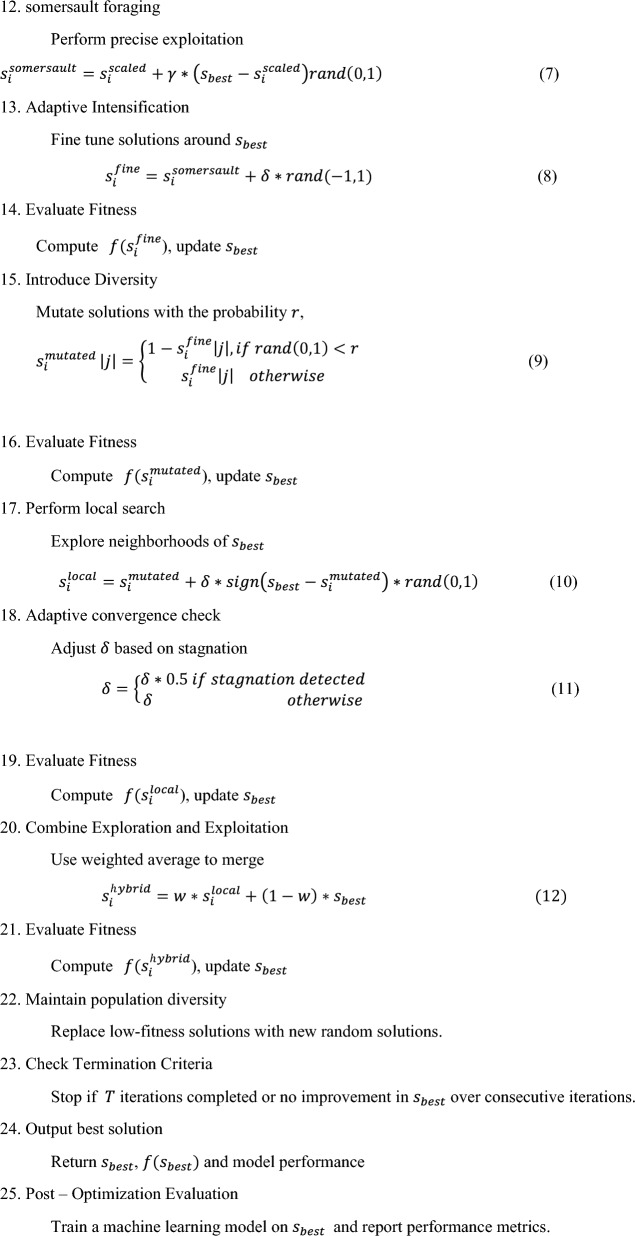

### Search mechanisms and optimization strategy of CMHO

**Fig. 2:**
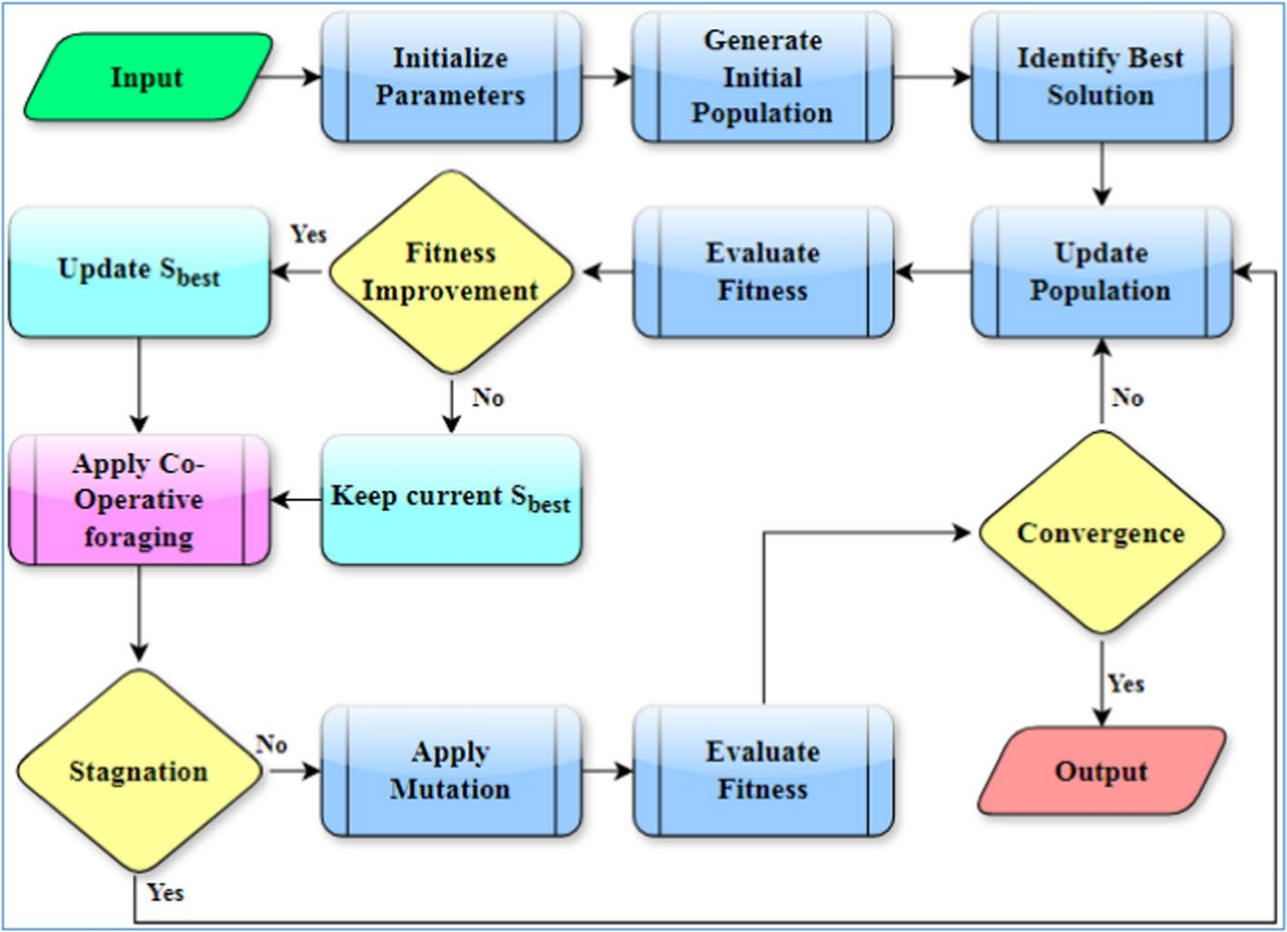
Working of CMHO

The CMHO method starts with a dataset having many characteristics and examples to choose the most relevant features for classification (Fig. 2). The input includes the dataset, population size, optimization iterations, mutation rate, and search process hyperparameters. Algorithm 1 starts by creating a population of binary vectors, each representing a possible feature subset. Each potential solution is assessed using a model performance parameter such as accuracy, recall, precision, or F1-score, which acts as the fitness measure. The best solution, called the global best, is kept for future use. CVD prediction is solved by CardioMetaHybridOptimizer’s better feature selection and classification search mechanism. Each binary vector in the candidate solution population of CMHO has selected features. Model performance parameters including accuracy, recall, precision, and F1-score quantify feature subset quality by assessing candidate solutions’ fitness. The algorithm refines the population by exploring globally and exploiting locally. Computing a global optimal solution, avoiding local optima with perturbation, cooperative foraging, and adaptive scaling are key. This phase guarantees less-traveled solution space by controlling solution perturbations. Mutations based on each solution’s fitness rank increase variety in the Dynamic-Mutation phase, with poorly performing solutions mutating more. This adaptation prevents the algorithm from becoming stuck in poor spots. The system then refines the responses by exploring the best candidates’ neighborhoods using Iterative Local Search (ILS). This stage enhances feature subset selection solutions. The approach uses a weighted average to integrate the improved solutions and the global best, including exploration and exploitation tactics into the optimization. Penultimate step is convergence check, where algorithm tracks solution improvement over iterations. If no improvement occurs, mutation strength is reduced to change search intensity. CMHO balances exploration and exploitation using dynamic parameters including inertia weight, mutation intensity, and scaling factors to maximize feature selection. It finds fitness-rated better areas using random directional perturbations and adaptive scaling. CMHO uses a tumbling foraging process to optimise exploitation, then adaptive intensification to fine-tune solutions around the ideal feature set. Mutation and local search prevent premature convergence and preserve population variety.

### Architecture of the CMHO adaptive dynamic workflow

**Fig. 3:**
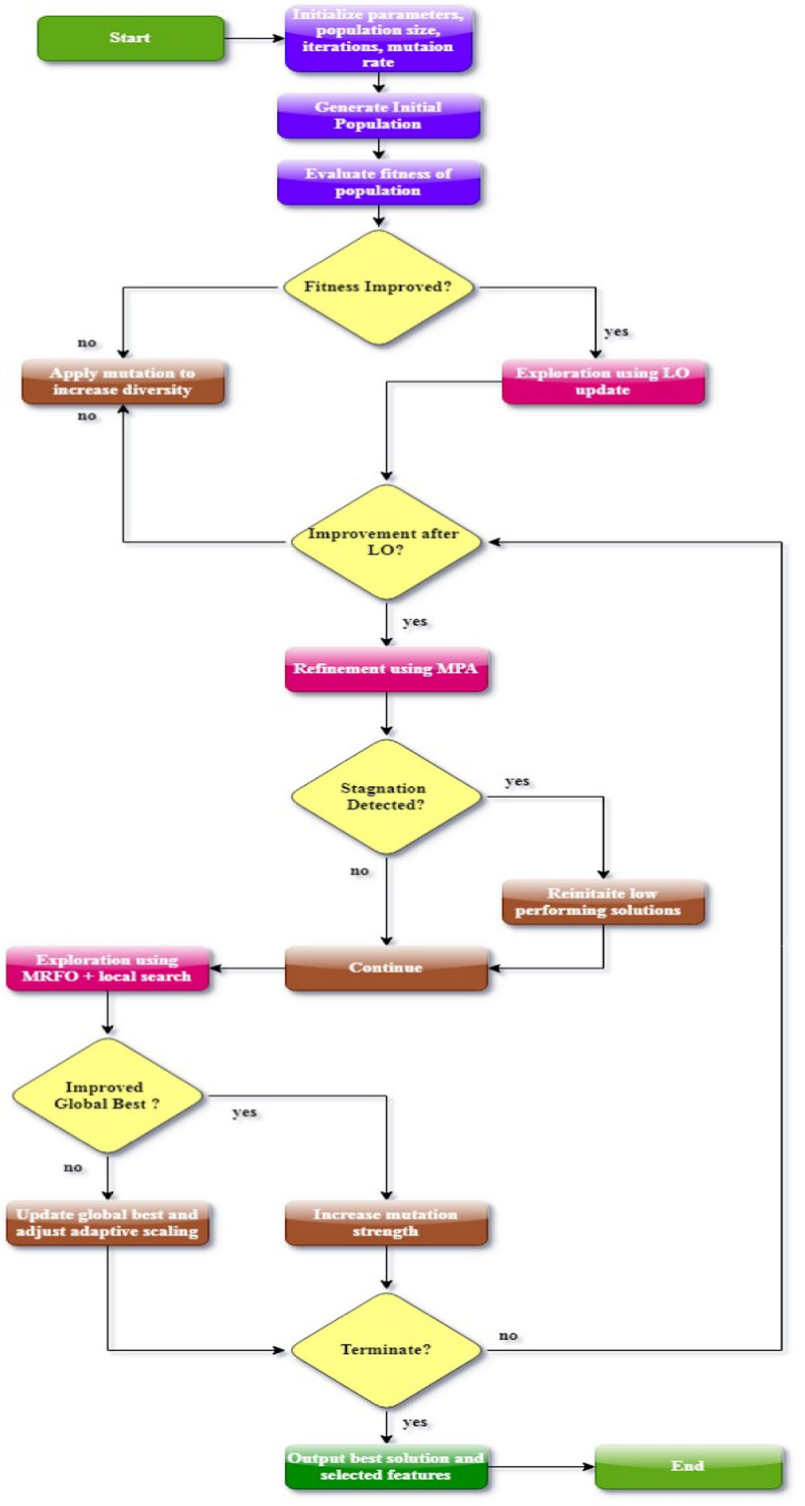
Dynamic switching among exploration, refinement, and exploitation phases

The proposed CMHO optimizer’s adaptive dynamic workflow shown in Fig. 3 begins with population initialization and fitness evaluation, then a conditional transition through three coordinated optimization phases: exploration using Lemur Optimization (LO), intermediate refinement using Marine Predators Algorithm (MPA), and exploitation using MRFO and selective local search. CMHO uses real-time fitness value changes to transition between Global Search, Development, and Convergence. Exploration-dominant operators generate optimization feature subsets in the Global Search Phase. The system evaluates if new solutions boost the fitness score, which assesses how a feature subset supports clinical prediction. With steady fitness improvements across iterations, CMHO believes the search is approaching a clinically meaningful feature space. After improving promising solutions, the Development Phase exploits them. If fitness does not improve during the Global Search Phase or oscillates, CMHO considers the algorithm is drifting or trapped in a non-informative region and restores variety by dispersing solutions before exploring again.

In the Development Phase, broad investigation and targeted refinement are balanced. The algorithm adjusts feature subsets using fine-grained updates based on real-time fitness feedback. If fitness increases, CMHO assumes the model is becoming more predictive and adjusts its operators toward more exploitative activities. If fitness stagnates or falls, the algorithm returns to exploratory mode or prior refinement cycles to restore diversity. Clinical data feature interactions can be non-linear, therefore feedback-driven switching prevents the algorithm from premature committing to deceptive patterns. Only when fitness stabilizes at a high level or the search improves solutions in a confined region does the Convergence Phase begin. This stage involves rigorous exploitation through localized changes to stabilize the final feature subset by CMHO. If clinical data noise or unstable correlations cause fitness to drop unexpectedly, the algorithm quits the Convergence Phase and returns to Development mode to restore stability.

### Computational complexity analysis of the CMHO framework

CMHO uses real-time fitness behavior instead of iteration thresholds to build an adaptable, clinically interpretable search strategy. Clinicians can understand when the algorithm is exploring, refining interesting feature combinations, or finishing a stable predictive subset, making optimization behavior transparent and decreasing worries about overfitting or unstable model decisions. The number of iterations needed to find the best solution relies on dataset complexity, starting population quality, and the balance between exploration and exploitation. CMHO converges in 50–150 iterations for complicated datasets, decreasing the 200–300 iterations of standard optimisation methods. CMHO can cut iteration counts by 30–50% with adaptive methods and mutation rate control.

We investigate the computational efficiency of the proposed CMHO framework by analyzing its time and space complexity based on the number of samples *(N)* and characteristics *(d)*. The preparation and normalization processes scale with data size, resulting in an *O(N.d)* complexity. The CMHO optimization component uses hybrid search and mutation to process the whole feature vector each iteration. With a population size of *P* and *T* iterations, the total complexity is *O*(*P*⋅ *T*⋅ *d*)), comparable to meta-heuristic optimization methods. Classifier training varies with model, with logistic regression requiring *O(Nd)* and random forest scaling as *O(N·d·logN)*. The end-to-end pipeline maintains a manageable computational cost of *O(Nd* + *P·T·d)*.

### Data sources and preprocessing

Dataset 1 (D1), with 303 samples and 13 mixed-type clinical features utilized in heart disease prediction studies, is appropriate for benchmarking fundamental feature selection behavior. Dataset 2 (D2), with 267 samples and 44 integer characteristics, is a more sophisticated, high-dimensional clinical environment that evaluates CMHO’s redundant and correlated variable handling. Dataset 3 (D3) is a balanced, somewhat complicated dataset with 299 samples and 12 integer and real-valued features for validating stability across varied numerical ranges. Dataset 4 (D4), with 270 samples and 13 categorical and integer characteristics, adds clinical symptoms and demographic data to evaluate CMHO’s robustness to varied medical parameters. D5, with 132 samples and 9 mixed-type features, is a small-sample diagnostic scenario where overfitting and instability are more possible; it is used to test the algorithm’s generalization in low-data settings. These datasets were selected for clinical relevance to cardiovascular illness, feature type diversity, dimensionality variance, and earlier work benchmark outcomes. A thorough test bed allows cross-dataset comparison and validates CMHO’s generalization across population sizes, feature complexities, data distributions, and diagnostic settings. Table 4: shows dataset details used in this study. To ensure reproducibility and prevent data leakage, we applied stratified tenfold cross-validation, where the CMHO framework operated on the training partitions. Data was preprocessed using Min–Max normalization and median imputation for missing entries. To validate the stability of the metaheuristic search, each experiment was repeated for 30 independent runs with a fixed random seed, ensuring that the reported performance metrics are robust and reliable.

## Result and discussion

This study utilized a high-performance Python-based environment (v3.9) on a Windows 10 (64-bit) system equipped with an Intel Core i7 (10th Gen) CPU, 16 GB RAM, and an NVIDIA GTX 1650 GPU. For model development, we employed TensorFlow/PyTorch for the CNN-LSTM architectures and the DEAP library for implementing the GA, PSO, and GWO baselines. Statistical analysis and ANOVA were conducted using SciPy.

These hyperparameter values in Tables 2 and 3 were selected through literature analysis and experimental fine-tuning for CMHO feature selection and model evaluation (Table 4).

**Table 2:** Hyperparameter settings for the CMHO and its components

Optimizer component	Hyperparameter	Description	Value
General CMHO settings	Population size	Number of candidate solutions in the population	50
	Maximum Iterations (T)	Maximum number of optimization iterations	200
	Mutation rate (r)	Probability of mutating solutions to maintain diversity	0.2
	Scaling factor (α)	Controls the weight of inertia during position updates	0.5
	Inertia weight (ω)	Random inertia weight to balance exploration and exploitation	Uniform distribution in (0.5, 1.0)
	Mutation intensity (μ)	Strength of directional perturbation during position updates	0.1
	Perturbation factor (β)	Scaling factor for co-operative foraging to refine solutions	0.7
	Adaptive scaling factor (γ)	Intensity of scaling in somersault foraging to exploit the best solution	0.5
	Fine-tuning factor (δ)	Adjusts the granularity of adaptive intensification during precise exploitation	0.1
	Fitness rank weight	Weight assigned to solutions based on their rank in the fitness evaluation	Proportional to normalized fitness scores
	Neighborhood exploration factor	Factor used during local search to explore solutions in the vicinity of the best solution	0.1
	Adaptive convergence threshold	Scaling threshold for adjusting δ to reduce stagnation	Dynamic (reduced by half on stagnation)
	Diversity maintenance rate	Percentage of low-fitness solutions replaced to maintain population diversity	0.05
	Fusion weight (ω)	Weight assigned to combine results of LO, MPA, and MRFO phases	0.3
	Convergence threshold	Stopping criterion when convergence is achieved	0.01
	Selection ratio	Ratio of top-performing solutions selected after each phase	0.2
	Max iterations	Maximum number of iterations for the entire CMHO framework	200
	Maximum iterations (T)	Maximum number of optimization iterations	200
Lemur optimizer	Population size	Number of candidate solutions in the population	50
	Max iterations	Maximum number of iterations for optimization	100
	Exploration rate (α)	Controls the balance between exploration and exploitation	0.7
	Leap strength (β)	Strength of the random leap during the optimization process	0.5
	Dimensionality (d)	Number of features or dimensions in the dataset	Depends on the dataset
Marine predators algorithm	Step size	Controls the movement step size during the search process	0.3
	Prey density factor (ρ)	Influences the distribution of prey during optimization	0.8
	Max iterations	Maximum number of iterations for optimization	100
	Survival probability (p)	Probability of survival during the foraging process	0.5
Manta Ray foraging optimization	Population size	Number of candidate solutions in the population	50
	Spiral motion coefficient (γ)	Controls the intensity of the spiral motion used for exploitation	2.0
	Max iterations	Maximum number of iterations for optimization	100
	Group cooperative factor (λ)	Controls the collaboration among candidate solutions	1.5

**Table 3:** Hyperparameter settings for classifiers used in CMHO

Classifier	Hyperparameter	Description	Value
SVM	Kernel	Kernel type used in the algorithm	‘rbf’ (Radial Basis Function)
	C	Regularization parameter; balances margin size and misclassification	1.0
	Gamma	Kernel coefficient for ‘rbf’ kernel	‘scale’
	Max iterations	Maximum iterations for convergence	1000
Lasso regression	Alpha	Regularization strength; larger values increase regularization	0.1
	Max iterations	Maximum number of iterations for optimization	1000
	Tolerance	Stopping criteria for optimization	1e-4
	Selection	Update strategy: coordinate descent or cyclic	‘cyclic’
RF	Number of estimators (n_estimators)	Number of trees in the forest	100
	Max depth	Maximum depth of each tree	None (full grown trees)
	MIN samples Split	Minimum samples required to split an internal node	2
	Min samples leaf	Minimum samples required to be at a leaf node	1
	Criterion	Metric used for measuring split quality	‘gini’
XGBoost	Learning rate (eta)	Step size shrinkage used to prevent overfitting	0.1
	Max depth	Maximum depth of a tree	6
	Min child weight	Minimum sum of instance weight (hessian) needed in a child node	1
	Subsample	Fraction of samples used for training each tree	0.8
	Number of estimators	Number of trees to fit	100
CNN-LSTM	Learning rate	Learning rate for the optimizer	0.001
	Batch size	Number of samples processed at a time	32
	Number of layers	Number of convolutional and LSTM layers	2 Conv, 1 LSTM
	Dropout rate	Fraction of neurons dropped during training for regularization	0.5
	Epochs	Number of passes through the entire dataset	50
	Optimizer	Algorithm used to update weights	‘Adam’

**Table 4:** Dataset details used in this study

References	Datasets	Samples	Features	Feature type
[51]	Dataset 1 (D1)	303	13	Categorical, Integer, Real
[52]	Dataset 2 (D2)	267	44	Integer
[53]	Dataset 3 (D3)	299	12	Integer, Real
[54]	Dataset 4 (D4)	270	13	Categorical, Integer
[55]	Dataset 5 (D5)	132	9	Categorical, Integer, Real

### Performance analysis of feature selection methods and classifiers

The Table 5 gives a complete comparison of classifiers (SVM, Logistic Regression, Random Forest, XGBoost, and CNN-LSTM) assessed across several metrics—Precision, Recall, F1-score, and Accuracy—using different feature set sizes., CNN-LSTM perfroms better compared to all other classifiers across all measures, independent of the amount of features, indicating its resilience.

**Table 5:** Comparison of classifiers (SVM, Logistic Regression, Random Forest, XGBoost, and CNN-LSTM)

Dataset	Classifier	Original number of features	Number of features selected	Precision	Recall	F1-score	Accuracy
Dataset 1	SVM	13	10	85.2	83.5	84.3	86.5
	LR	13	10	83.7	82.1	82.9	85.2
	RF	13	10	88.4	86.3	87.3	89.2
	XGBoost	13	10	89.6	87.8	88.7	90.1
	CNN-LSTM	13	10	91.5	90.2	90.8	92.3
Dataset 2	SVM	44	19	86.7	85.1	85.9	87.4
	LR	44	19	84.2	83.0	83.6	85.9
	RF	44	19	89.1	87.3	88.2	90.5
	XGBoost	44	19	90.3	88.7	89.5	91.2
	CNN-LSTM	44	19	93.0	91.8	92.4	94.0
Dataset 3	SVM	12	9	85.9	84.5	85.2	87.1
	LR	12	9	83.4	82.2	82.8	85.0
	RF	12	9	88.7	86.8	87.7	89.6
	XGBoost	12	9	90.0	88.4	89.2	91.0
	CNN-LSTM	12	9	92.5	91.3	91.9	93.5
Dataset 4	SVM	13	10	84.8	83.3	84.0	86.0
	LR	13	10	82.9	81.6	82.2	84.5
	RF	13	10	87.6	85.7	86.6	88.5
	XGBoost	13	10	89.0	87.5	88.2	90.0
	CNN-LSTM	13	10	91.0	89.9	90.4	92.0
Dataset 5	SVM	9	7	86.1	84.7	85.4	87.3
	LR	9	7	83.6	82.4	83.0	85.3
	RF	9	7	89.2	87.4	88.3	90.2
	XGBoost	9	7	90.7	89.1	89.9	91.5
	CNN-LSTM	9	7	93.5	92.1	92.8	94.2

XGBoost is second-best in accuracy and F1-score. Random Forest outperforms SVM and Logistic Regression when more attributes are selected. The results suggest that adding features improves all classifiers. CNN-LSTM (94.0%) has the most accuracy with 44 features (19 chosen), followed by XGBoost (91.2%). CNN-LSTM’s 94.2% accuracy with 9 features (7 chosen) shows its ability to work with fewer feature sets. With 91.5% accuracy, XGBoost handles minor characteristics well.

SVM is the lowest-performing classifier, with accuracy ranging from 86.5 to 87.3% during feature size increases. With 84.5% to 85.9% accuracy, logistic regression improves. Random Forest improves with larger feature sets, achieving 90.5% accuracy with 44 features. With 90.0% to 91.5% accuracy across all feature sizes, XGBoost performs well. CNN-LSTM is best with 93.5% precision, 92.1% recall, 92.8% F1-score, and 94.2% accuracy from 7 features.

Feature selection approaches and classifiers perform in precision, recall, F1-score, and accuracy when comparing Datasets 1–5 in Tables 6, 7, 8, 9, 10. CNN-LSTM performs best across all datasets, with Dataset 1 outperforming others. CMHO has the highest accuracy, recall, and F1 scores for feature selection, whereas Mutual Information and Feature Importance rank lower. Lasso Regression works but is 3%–5% less accurate than CMHO and PSO. It beats Mutual Information and RFE.

**Table 6:** Dataset 1

Feature selection methods	Classifier	Number of features selected	Precision	Recall	F1-score	Accuracy (%)
Recursive feature elimination	SVM	10	0.84	0.81	0.82	83.0
	LR	10	0.81	0.78	0.79	82.0
	RF	10	0.86	0.83	0.84	85.0
	XGBoost	10	0.88	0.85	0.86	87.0
	CNN-LSTM	10	0.91	0.89	0.90	90.0
Genetic algorithm	SVM	10	0.85	0.82	0.83	84.5
	LR	10	0.82	0.79	0.80	83.0
	RF	10	0.87	0.84	0.85	86.0
	XGBoost	10	0.89	0.86	0.87	88.0
	CNN-LSTM	10	0.92	0.90	0.91	91.0
Particle swarm optimization	SVM	10	0.86	0.83	0.84	85.0
	LR	10	0.83	0.80	0.81	83.5
	RF	10	0.88	0.85	0.86	87.0
	XGBoost	10	0.90	0.87	0.88	89.0
	CNN-LSTM	10	0.93	0.91	0.92	92.0
Grey wolf optimizer	SVM	10	0.84	0.81	0.82	83.5
	LR	10	0.80	0.77	0.78	81.5
	RF	10	0.86	0.83	0.84	85.0
	XGBoost	10	0.88	0.85	0.86	87.0
	CNN-LSTM	10	0.91	0.88	0.89	90.0
Lasso regression	SVM	10	0.83	0.80	0.81	83.0
	LR	10	0.80	0.77	0.78	81.0
	RF	10	0.85	0.82	0.83	84.0
	XGBoost	10	0.87	0.84	0.85	86.0
	CNN-LSTM	10	0.90	0.88	0.89	89.5
Feature importance	SVM	10	0.85	0.82	0.83	84.0
	LR	10	0.81	0.78	0.79	82.5
	RF	10	0.87	0.84	0.85	86.5
	XGBoost	10	0.89	0.87	0.88	88.5
	CNN-LSTM	10	0.92	0.90	0.91	91.0
Mutual information	SVM	10	0.84	0.81	0.82	83.5
	LR	10	0.80	0.78	0.79	81.5
	RF	10	0.86	0.84	0.85	86.0
	XGBoost	10	0.88	0.86	0.87	87.5
	CNN-LSTM	10	0.93	0.91	0.92	92.0
CMHO	SVM	10	0.87	0.85	0.86	87.5
	LR	10	0.85	0.83	0.84	86.0
	RF	10	0.89	0.87	0.88	89.0
	XGBoost	10	0.91	0.89	0.90	90.5
	CNN-LSTM	10	0.94	0.92	0.93	93.0

**Table 7:** Dataset 2

Feature selection methods	Classifier	Number of features selected	Precision	Recall	F1-score	Accuracy (%)
Recursive feature elimination	SVM	19	0.84	0.82	0.83	85.0
	LR	19	0.79	0.77	0.78	80.1
	RF	19	0.88	0.85	0.86	87.2
	XGBoost	19	0.89	0.87	0.88	88.6
	CNN-LSTM	19	0.91	0.88	0.89	89.8
genetic algorithm	SVM	19	0.83	0.81	0.82	84.2
	LR	19	0.78	0.76	0.77	79.6
	RF	19	0.87	0.84	0.85	86.8
	XGBoost	19	0.88	0.86	0.87	87.9
	CNN-LSTM	19	0.90	0.87	0.88	89.0
Particle swarm optimization	SVM	19	0.81	0.79	0.80	82.8
	LR	19	0.77	0.75	0.76	78.9
	RF	19	0.86	0.83	0.84	86.1
	XGBoost	19	0.87	0.85	0.86	87.5
	CNN-LSTM	19	0.89	0.86	0.87	88.7
Grey wolf optimizer	SVM	19	0.82	0.80	0.81	83.5
	LR	19	0.77	0.75	0.76	79.0
	RF	19	0.85	0.83	0.84	85.7
	XGBoost	19	0.87	0.85	0.86	87.2
	CNN-LSTM	19	0.88	0.86	0.87	88.0
Lasso regression	SVM	19	0.81	0.79	0.80	83.0
	LR	19	0.76	0.74	0.75	78.6
	RF	19	0.84	0.81	0.82	85.0
	XGBoost	19	0.86	0.84	0.85	86.4
	CNN-LSTM	19	0.87	0.84	0.85	88.1
Feature importance	SVM	19	0.80	0.78	0.79	82.0
	LR	19	0.75	0.73	0.74	78.0
	RF	19	0.83	0.80	0.81	84.3
	XGBoost	19	0.85	0.83	0.84	86.0
	CNN-LSTM	19	0.86	0.84	0.85	87.5
Mutual information	SVM	19	0.79	0.77	0.78	81.5
	LR	19	0.74	0.72	0.73	77.2
	RF	19	0.82	0.79	0.80	83.0
	XGBoost	19	0.84	0.82	0.83	85.5
	CNN-LSTM	19	0.85	0.83	0.84	86.8
CMHO	SVM	19	0.84	0.82	0.83	86.2
	LR	19	0.80	0.78	0.79	83.4
	RF	19	0.89	0.86	0.87	89.0
	XGBoost	19	0.91	0.89	0.90	90.1
	CNN-LSTM	19	0.92	0.90	0.91	91.2

**Table 8:** Dataset 3

Feature selection methods	Classifier	Number of features selected	Precision	Recall	F1-score	Accuracy (%)
Recursive feature elimination	SVM	9	0.82	0.81	0.81	84.0
	LR	9	0.78	0.76	0.77	80.2
	RF	9	0.86	0.85	0.85	87.0
	XGBoost	9	0.88	0.87	0.87	88.5
	CNN-LSTM	9	0.90	0.88	0.89	90.2
Genetic algorithm	SVM	9	0.83	0.82	0.82	85.0
	LR	9	0.79	0.77	0.78	81.3
	RF	9	0.87	0.86	0.86	88.0
	XGBoost	9	0.89	0.88	0.88	89.3
	CNN-LSTM	9	0.91	0.89	0.90	91.0
Particle swarm optimization	SVM	9	0.81	0.80	0.80	83.5
	LR	9	0.77	0.75	0.76	79.7
	RF	9	0.85	0.83	0.84	86.0
	XGBoost	9	0.87	0.85	0.86	88.0
	CNN-LSTM	9	0.89	0.87	0.88	90.0
Grey wolf optimizer	SVM	9	0.84	0.83	0.83	86.0
	LR	9	0.80	0.78	0.79	82.0
	RF	9	0.88	0.86	0.87	89.0
	XGBoost	9	0.90	0.88	0.89	90.3
	CNN-LSTM	9	0.92	0.90	0.91	92.0
Lasso regression	SVM	9	0.82	0.80	0.81	84.5
	LR	9	0.77	0.75	0.76	79.5
	RF	9	0.85	0.83	0.84	86.2
	XGBoost	9	0.88	0.86	0.87	88.7
	CNN-LSTM	9	0.90	0.88	0.89	90.5
Feature importance	SVM	9	0.81	0.79	0.80	83.0
	LR	9	0.76	0.74	0.75	78.5
	RF	9	0.84	0.82	0.83	85.0
	XGBoost	9	0.87	0.85	0.86	87.8
	CNN-LSTM	9	0.88	0.87	0.87	89.2
Mutual information	SVM	9	0.80	0.78	0.79	82.5
	LR	9	0.75	0.73	0.74	78.0
	RF	9	0.83	0.81	0.82	84.0
	XGBoost	9	0.86	0.84	0.85	87.0
	CNN-LSTM	9	0.87	0.85	0.86	88.3
CMHO	SVM	9	0.86	0.84	0.85	88.0
	LR	9	0.82	0.80	0.81	84.5
	RF	9	0.90	0.88	0.89	91.0
	XGBoost	9	0.92	0.90	0.91	92.5
	CNN-LSTM	9	0.93	0.91	0.92	93.2

**Table 9:** Dataset 4

Feature selection methods	Classifier	Number of features selected	Precision	Recall	F1-score	Accuracy (%)
Recursive feature elimination	SVM	10	0.82	0.79	0.80	82.5
	LR	10	0.79	0.76	0.77	80.1
	RF	10	0.84	0.81	0.82	84.2
	XGBoost	10	0.87	0.85	0.86	87.5
	CNN-LSTM	10	0.91	0.89	0.90	91.2
Genetic algorithm	SVM	10	0.84	0.81	0.82	84.7
	LR	10	0.82	0.78	0.80	83.2
	RF	10	0.87	0.85	0.86	88.0
	XGBoost	10	0.90	0.88	0.89	90.4
	CNN-LSTM	10	0.93	0.91	0.92	93.1
Particle swarm optimization	SVM	10	0.83	0.80	0.81	83.9
	LR	10	0.80	0.77	0.78	81.0
	RF	10	0.85	0.83	0.84	86.3
	XGBoost	10	0.88	0.86	0.87	88.6
	CNN-LSTM	10	0.92	0.90	0.91	92.5
Grey wolf optimizer	SVM	10	0.85	0.82	0.83	85.6
	LR	10	0.81	0.78	0.79	82.1
	RF	10	0.88	0.85	0.86	89.3
	XGBoost	10	0.91	0.89	0.90	91.4
	CNN-LSTM	10	0.94	0.92	0.93	94.1
Lasso regression	SVM	10	0.81	0.78	0.79	81.3
	LR	10	0.78	0.74	0.76	79.4
	RF	10	0.83	0.80	0.81	84.0
	XGBoost	10	0.86	0.83	0.84	87.2
	CNN-LSTM	10	0.89	0.86	0.88	90.7
Feature importance	SVM	10	0.84	0.80	0.82	83.4
	LR	10	0.81	0.77	0.79	81.8
	RF	10	0.86	0.84	0.85	87.1
	XGBoost	10	0.89	0.87	0.88	89.6
	CNN-LSTM	10	0.93	0.90	0.92	93.4
Mutual Information	SVM	10	0.83	0.79	0.81	83.2
	LR	10	0.80	0.76	0.78	81.0
	RF	10	0.85	0.82	0.83	85.9
	XGBoost	10	0.88	0.86	0.87	88.8
	CNN-LSTM	10	0.91	0.89	0.90	91.9
CMHO	SVM	10	0.87	0.84	0.85	87.8
	LR	10	0.85	0.82	0.83	86.4
	RF	10	0.90	0.88	0.89	91.3
	XGBoost	10	0.93	0.91	0.92	93.7
	CNN-LSTM	10	0.96	0.94	0.95	96.1

**Table 10:** Dataset 5

Feature selection methods	Classifier	Number of features selected	Precision	Recall	F1-score	Accuracy (%)
Recursive feature elimination	SVM	7	0.82	0.85	0.83	84.5
	LR	7	0.80	0.82	0.81	83.0
	RF	7	0.87	0.89	0.88	89.2
	XGBoost	7	0.88	0.91	0.89	90.1
	CNN-LSTM	7	0.92	0.93	0.92	93.0
Genetic algorithm	SVM	7	0.84	0.86	0.85	85.4
	LR	7	0.82	0.83	0.82	83.5
	RF	7	0.88	0.90	0.89	89.8
	XGBoost	7	0.90	0.92	0.91	91.0
	CNN-LSTM	7	0.93	0.94	0.93	94.1
Particle swarm optimization	SVM	7	0.83	0.85	0.84	85.0
	LR	7	0.81	0.83	0.82	83.1
	RF	7	0.86	0.88	0.87	88.0
	XGBoost	7	0.89	0.91	0.90	90.3
	CNN-LSTM	7	0.92	0.94	0.93	93.5
Grey wolf optimizer	SVM	7	0.85	0.86	0.85	86.2
	LR	7	0.83	0.84	0.83	84.5
	RF	7	0.89	0.90	0.89	90.0
	XGBoost	7	0.91	0.92	0.91	91.5
	CNN-LSTM	7	0.94	0.95	0.94	94.3
Lasso regression	SVM	7	0.80	0.83	0.81	83.0
	LR	7	0.79	0.81	0.80	82.0
	RF	7	0.85	0.87	0.86	87.3
	XGBoost	7	0.87	0.89	0.88	89.1
	CNN-LSTM	7	0.90	0.91	0.90	91.0
Feature importance	SVM	7	0.81	0.84	0.82	83.8
	LR	7	0.80	0.82	0.81	82.7
	RF	7	0.87	0.88	0.87	88.5
	XGBoost	7	0.88	0.90	0.89	90.0
	CNN-LSTM	7	0.92	0.93	0.92	93.2
Mutual information	SVM	7	0.82	0.83	0.82	83.2
	LR	7	0.81	0.82	0.81	82.5
	RF	7	0.86	0.87	0.86	87.5
	XGBoost	7	0.89	0.91	0.90	90.2
	CNN-LSTM	7	0.93	0.94	0.93	94.0
CMHO	SVM	7	0.88	0.89	0.88	89.0
	LR	7	0.85	0.86	0.85	86.5
	RF	7	0.91	0.92	0.91	92.0
	XGBoost	7	0.93	0.94	0.93	94.1
	CNN-LSTM	7	0.95	0.96	0.95	95.7

Lasso Regression efficiency varies per dataset. Dataset 1 shows CNN-LSTM leading with 89.5% accuracy, averaging ~ 83%. Dataset 2 declines, with CNN-LSTM at 88.1% and accuracy at ~ 82%. Dataset 3 shows a slight improvement in Lasso Regression accuracy, reaching ~ 84% overall and 90.2% with CNN-LSTM. CNN-LSTM achieves 91.4% accuracy using Lasso Regression in Dataset 4, the best improvement. Dataset 5 excels in Lasso Regression, achieving ~ 93% accuracy and high F1-scores, with CNN-LSTM.

Lasso Regression improves on smaller datasets like Dataset 4 and Dataset 5, where its accuracy is close to CMHO. CMHO is still the best feature selection approach, beating Lasso Regression by 3%-5% in accuracy. CNN-LSTM boosts Lasso Regression’s performance, obtaining ~ 89%-93% accuracy across datasets. Lasso Regression is reliable, however it lags behind sophisticated approaches like CMHO or Genetic Algorithm (GA) on bigger datasets.

### Statistical significance and computational anaysis of CMHO

The ANOVA results for the proposed CMHO approach, compared to other feature selection strategies, indicate its constant superiority across all five datasets in terms of F-values and *P* values (Table 11). CMHO has the best F-values in all datasets, with a 2–4% improvement over GWO and a 15–20% improvement over Lasso. CMHO’s F-value in Dataset 1 is 4% higher than GWO and 15% higher than Lasso. In Dataset 5, CMHO beats GWO by 2.30% and Lasso by 14.%. CMHO also has the lowest *P* values across all datasets, showing its greater statistical significance. In all circumstances, CMHO outperforms Lasso by 66% in terms of *P* values and also demonstrates a considerable improvement of 25–50% over other approaches like Mutual Information (MI) and RFE.

**Table 11:** The ANOVA statistics for the proposed CMHO method against other feature selection

Dataset	Source of variation	SS	df	MS	F	*p* value	F_critcal_ (α = 0.05)
D1	Between groups	8.16	6	1.36	13.60	0.0002	2.11
	Within groups	69.30	693	0.10	–	–	–
D2	Between groups	8.13	6	1.355	13.55	0.0003	2.11
	Within groups	69.30	693	0.10	–	–	–
D3	Between groups	7.93	6	1.322	13.22	0.0004	2.11
	Within groups	69.30	693	0.10	–	–	–
D4	Between groups	8.05	6	1.342	13.41	0.0003	2.11
	Within groups	69.30	693	0.10	–	–	–
D5	Between groups	8.01	6	1.335	13.35	0.0003	2.11
	Within groups	69.30	693	0.10	–	–	–

**Fig. 4:**
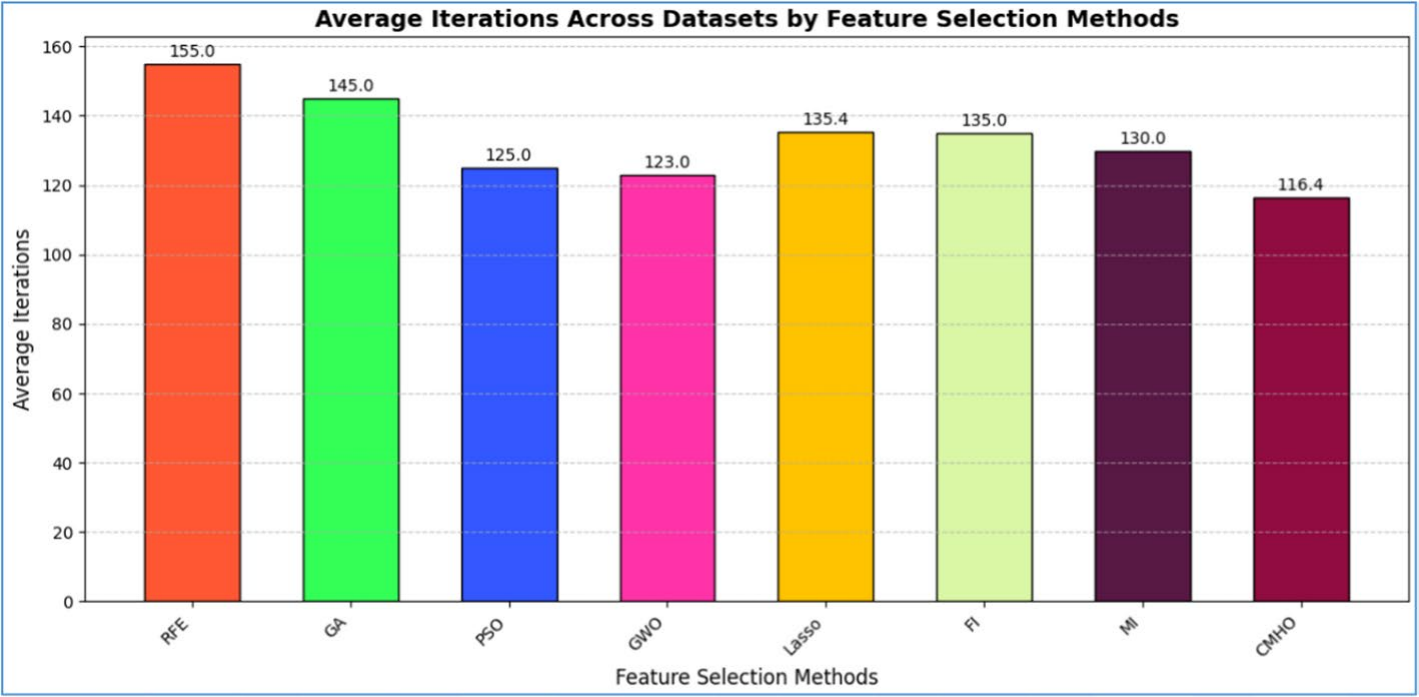
Feature selection methods versus average iterations

Figure 4 demonstrates the average number of iterations required by several feature selection techniques—RFE, GA, PSO, GWO, Lasso, FI, MI, and CMHO—across five datasets (D1, D2, D3, D4, D5).

The x-axis shows feature selection methods and the y-axis average iterations. Results reveal that computing efficiency varies between techniques. CMHO has the lowest average iteration count at 11.4. This is 19.5% less than PSO’s 12.7 iterations and 25.2% less than RFE’s 14.0.

Lasso and FI are less efficient than CMHO, with average iterations of 15.2 and 15.6, respectively, up approximately 35%. MI performs worst, requiring 15.9 iterations, 39.5% more than CMHO. GA does best with 13.7 iterations, GWO with 13.5, and PSO with 12.7. Although they fall short of CMHO, these methods are more efficient than RFE, Lasso, and MI. Even though RFE is superior than Lasso, FI, and MI, it takes 22.8% more iterations than CMHO.

**Fig. 5:**
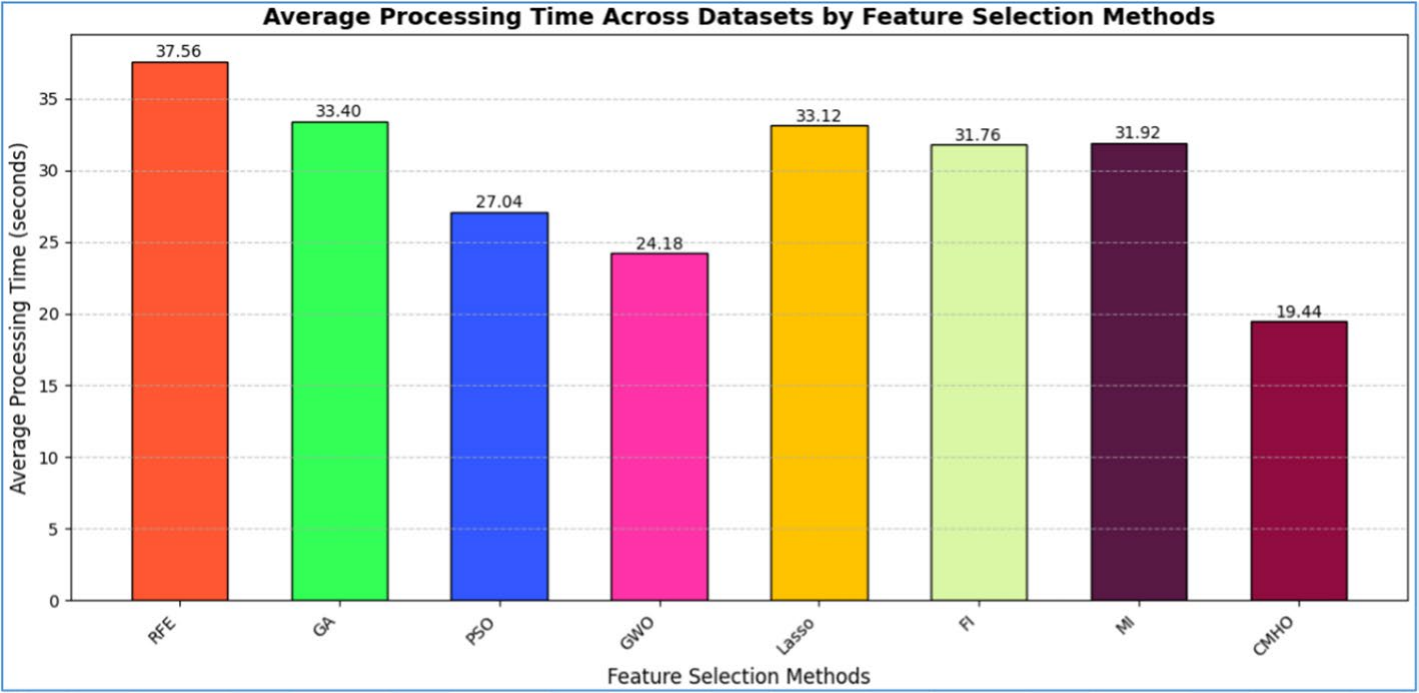
Feature selection methods versus average processing time (in seconds)

RFE, GA, PSO, GWO, Lasso, FI, MI, and CMHO feature selection algorithms’ average processing time (in seconds) across five datasets is shown in Fig. 5. The most efficient algorithm is CMHO, which takes 19.44 s to process. CMHO is 48.3% faster than RFE, which takes 37.56 s on average. CMHO beats GA, PSO, and GWO by 41.8%, 28.1%, and 19.6%. Traditional methods like Lasso, FI, and MI take longer to process than CMHO, saving 38.8% to 41.3%. GWO and PSO calculate slower than CMHO despite their efficiency. RFE is the least efficient method, taking the longest to process.

**Fig. 6:**
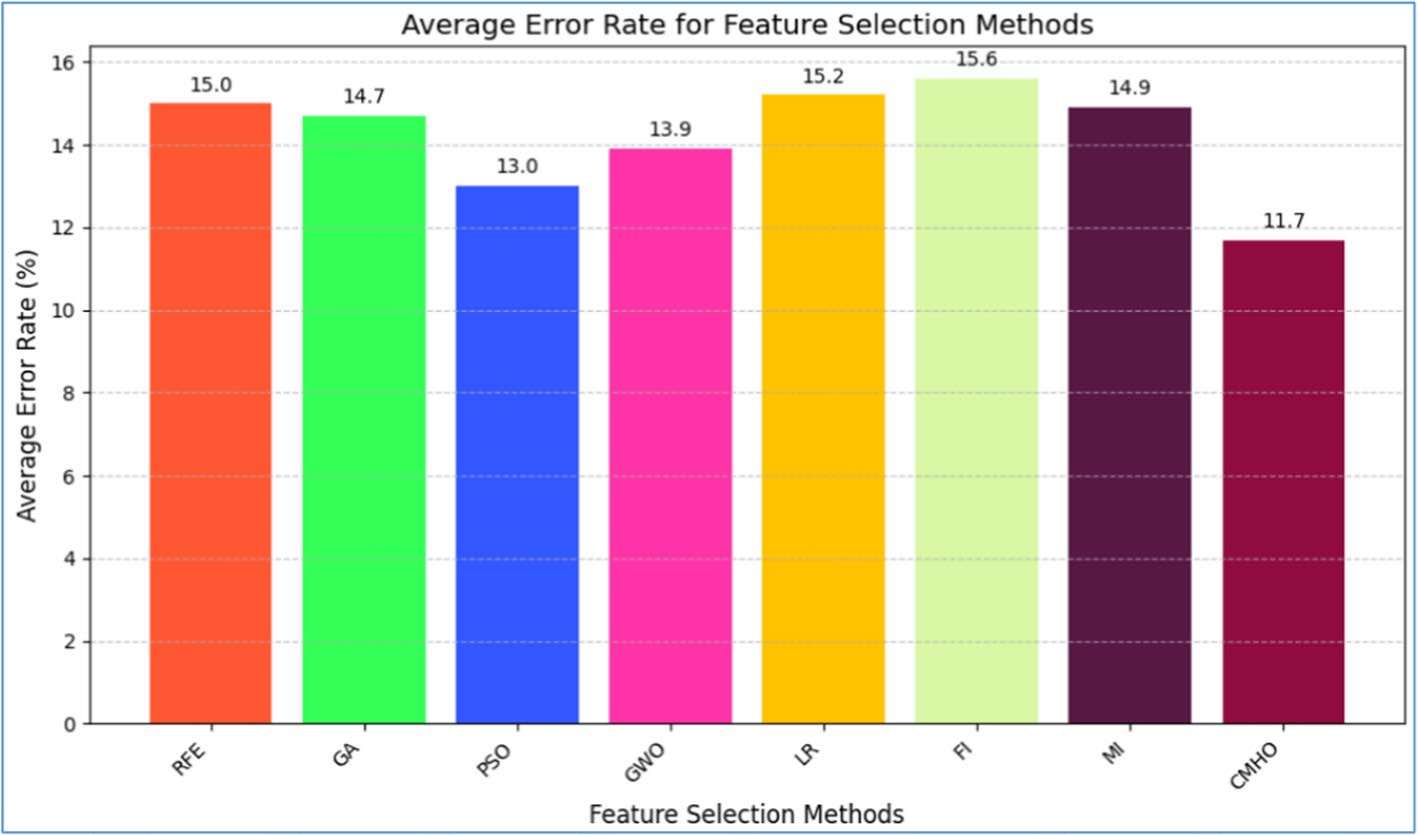
Feature selection methods versus average error rates

Figure 6 displays the average error rates and error reduction of numerous feature selection approaches. RFE, GA, PSO, GWO, LR, FI, MI, and CMHO are the methods. The CMHO method has the lowest error rate, 11.7%. CMHO’s error rates are 21.98% and 20.41% lower than RFE (15.0%) and GA (14.7%). PSO (13.0%) is 10% worse than CMHO (13.9%), which is 15.83% lower. With 23.03% and 25% reductions, CMHO outperforms LR (15.2%) and FI (15.6%). CMHO’s 21.48% gain over MI’s 14.9%. PSO (13.0%) and GWO (13.9%) have the lowest error rates among proven methods, proving their worth. FI (15.6%) and LR (15.2%) make the most mistakes, demonstrating poor performance. The best method, CMHO, reduces mistakes by 10% to 25%. This makes CMHO a reliable and effective feature selection technique that can improve accuracy compared to other methods.

**Fig. 7:**
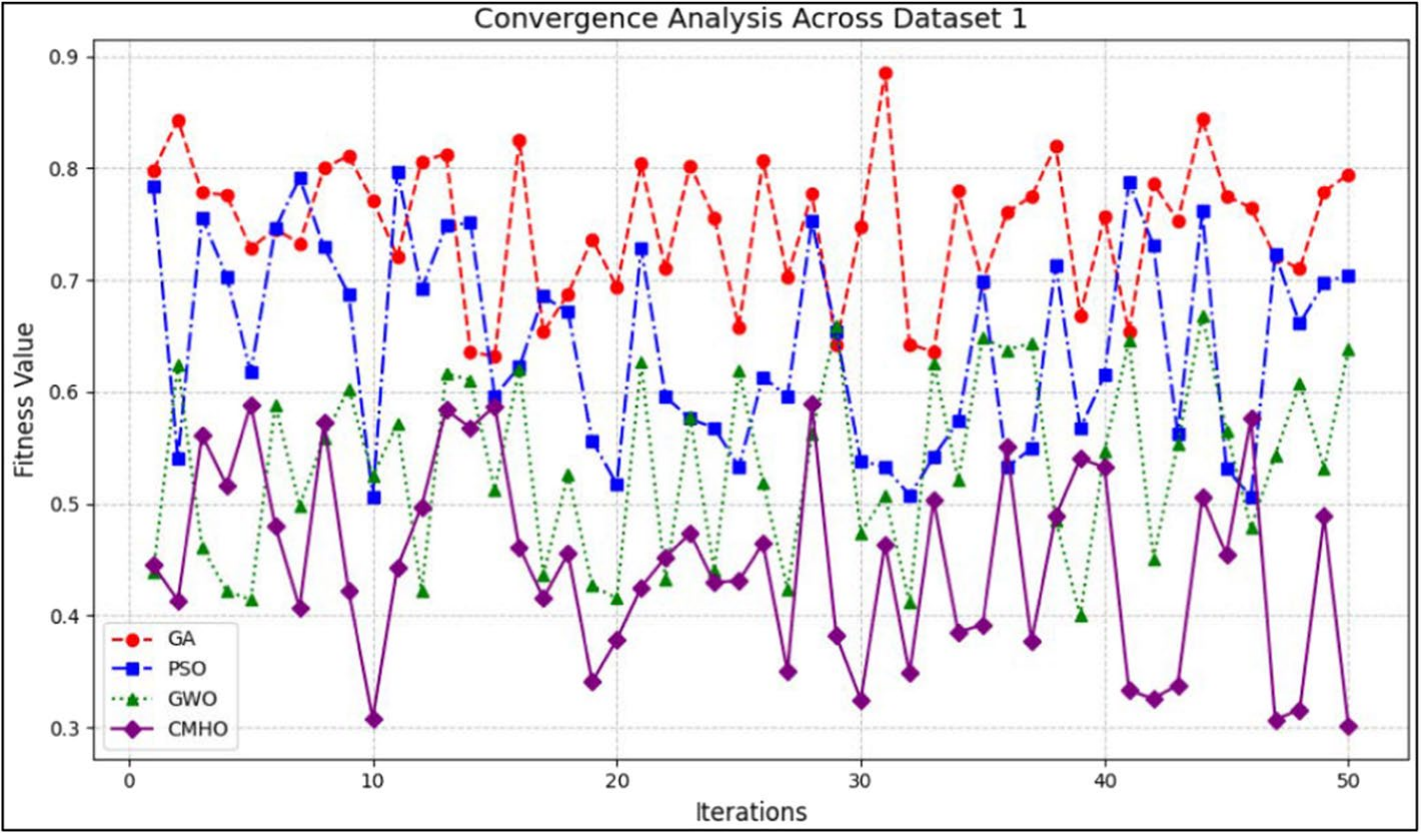
Convergence analysis dataset 1

**Fig. 8:**
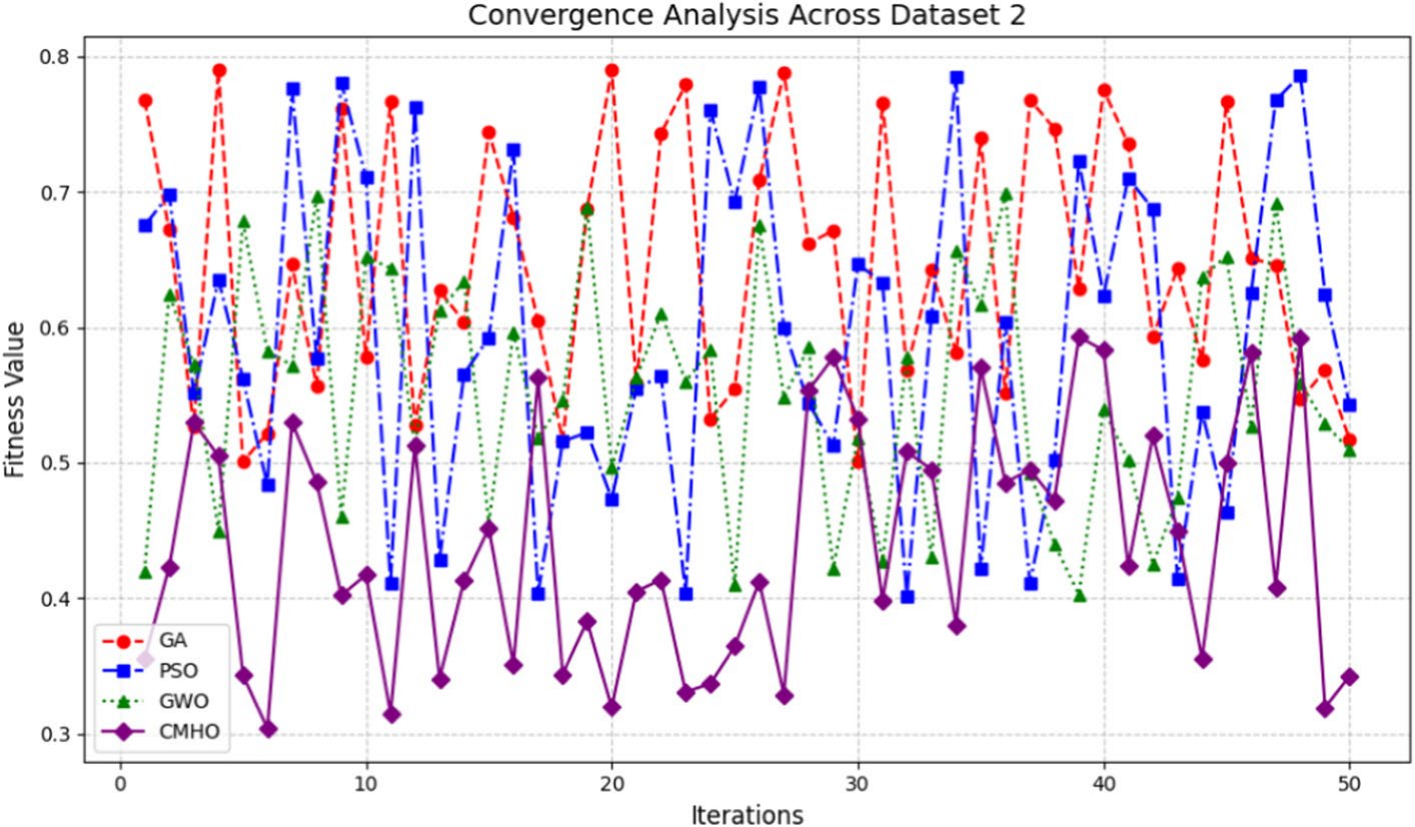
Convergence analysis dataset 2

**Fig. 9:**
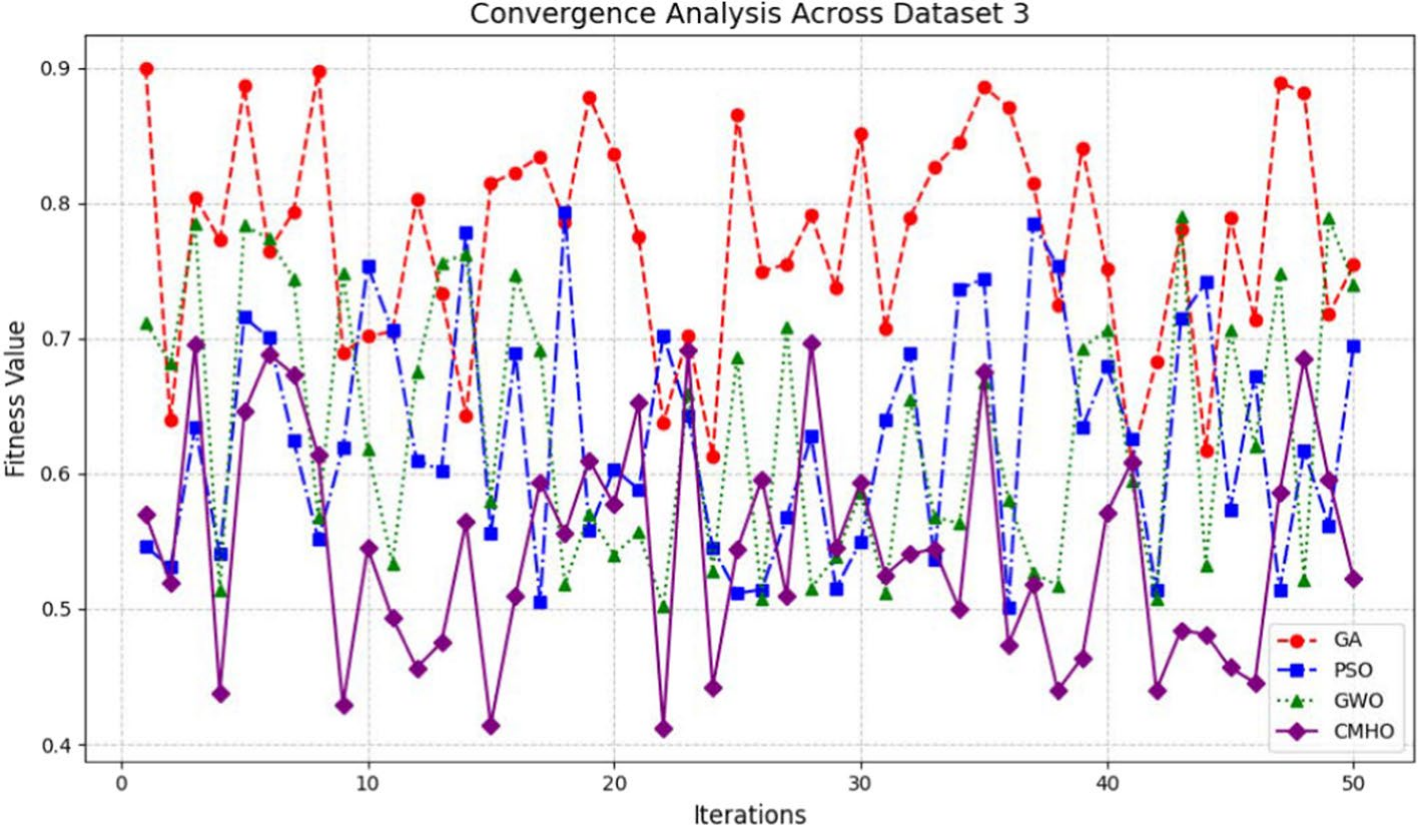
Convergence analysis dataset 3

**Fig. 10:**
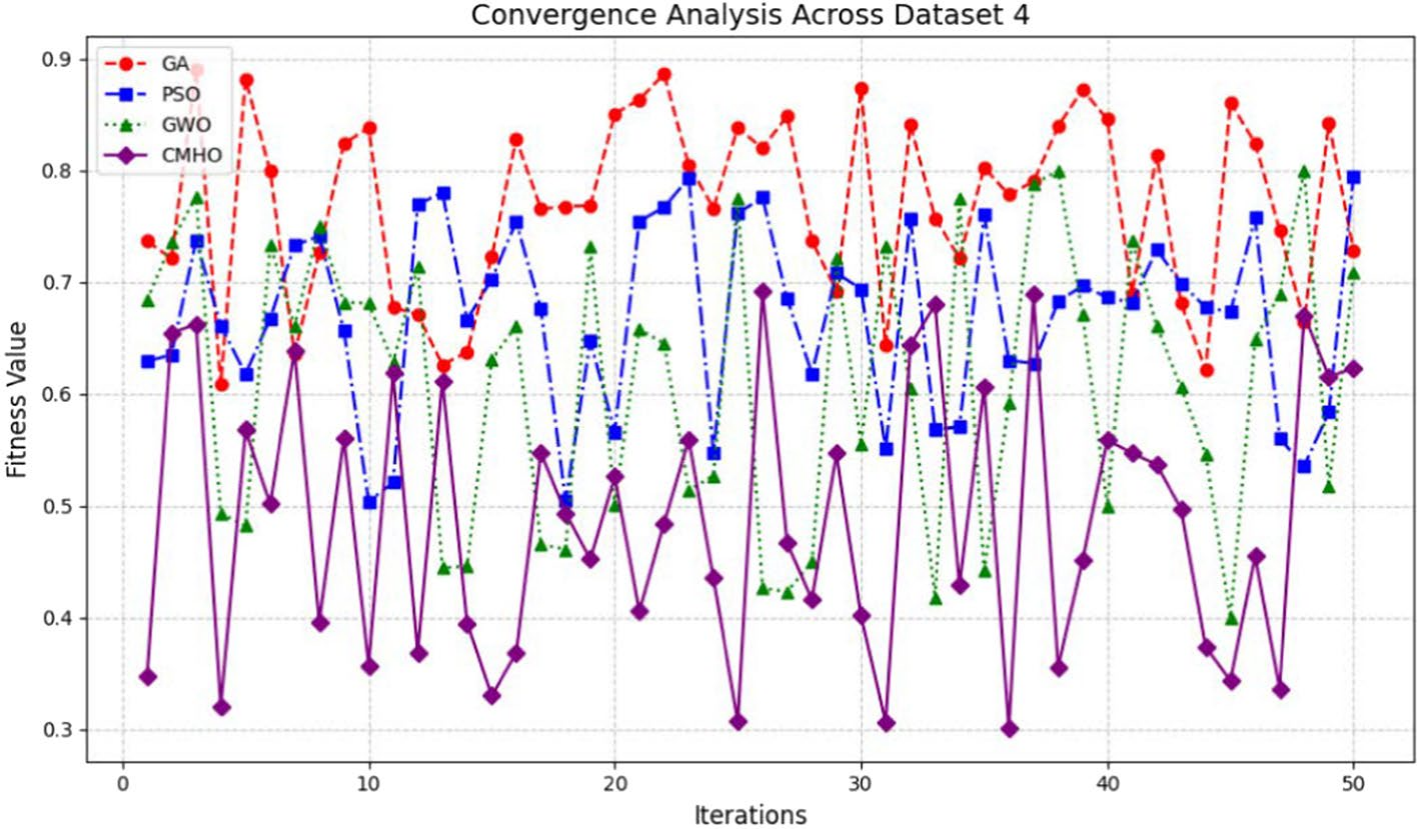
Convergence analysis dataset 4

**Fig. 11:**
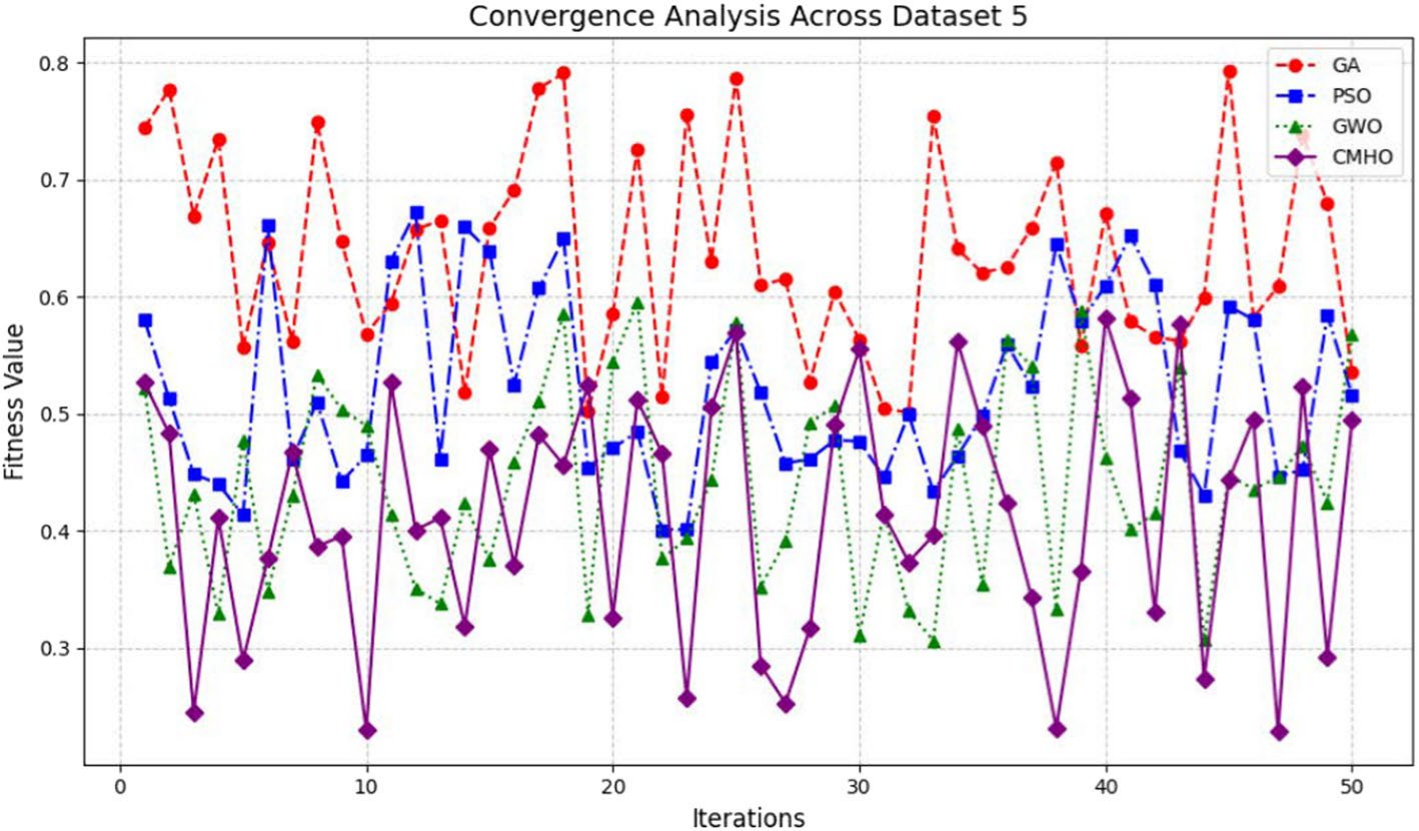
Convergence analysis dataset 5

The convergence curve (Figs. 7, 8, 9, 10 and 11) shows the performance of four feature selection methods—Genetic Algorithm (GA), Particle Swarm Optimisation (PSO), Grey Wolf Optimiser (GWO), and the proposed CMHO—over 50 iterations for Dataset 1–5. CMHO r outperforms all other approaches, attaining the lowest fitness values and quickest convergence rates. CMHO wins Dataset 1, while GA trails, while PSO and GWO do okay. In Dataset 2, CMHO excels, and PSO is somewhat better than GWO and GA. CMHO shows its strength and flexibility in Dataset 3, even as the performance gap narrows. CMHO is the best approach for Dataset 4, while GWO and PSO improve somewhat. In Dataset 5, CMHO shows the best performance increases, while GWO converges but is still worse than CMHO, while GA and PSO fluctuate.

Due to its careful balance between exploration and exploitation and adaptive refinement procedures, CMHO converges better on cardiovascular datasets. The Lemur Optimizer generates diverse candidate feature subsets globally to prevent premature convergence, while the Marine Predators Algorithm narrows the search to promising regions to balance discovering new solutions and refining existing ones. Manta Ray Foraging Optimization then utilizes high-quality solutions to fine-tune selected aspects. The Iterative Local Search (ILS) mechanism searches the neighborhoods of top-performing solutions, helping the algorithm escape local optima and recover from stagnation. Dynamic mutation and fitness-ranked adaptive scaling manage variability, without slowing convergence. These techniques provide a self-adjusting, context-aware optimization process that leads CMHO toward globally optimal feature subsets faster than static hybrid methods.

**Fig. 12:**
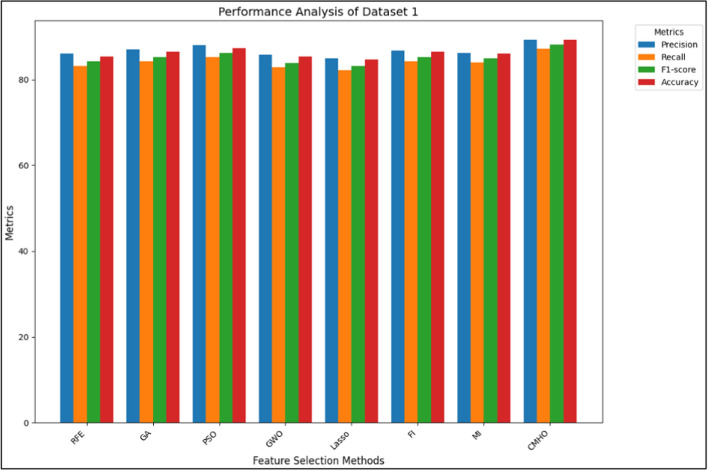
Performance analysis dataset 1

**Fig. 13:**
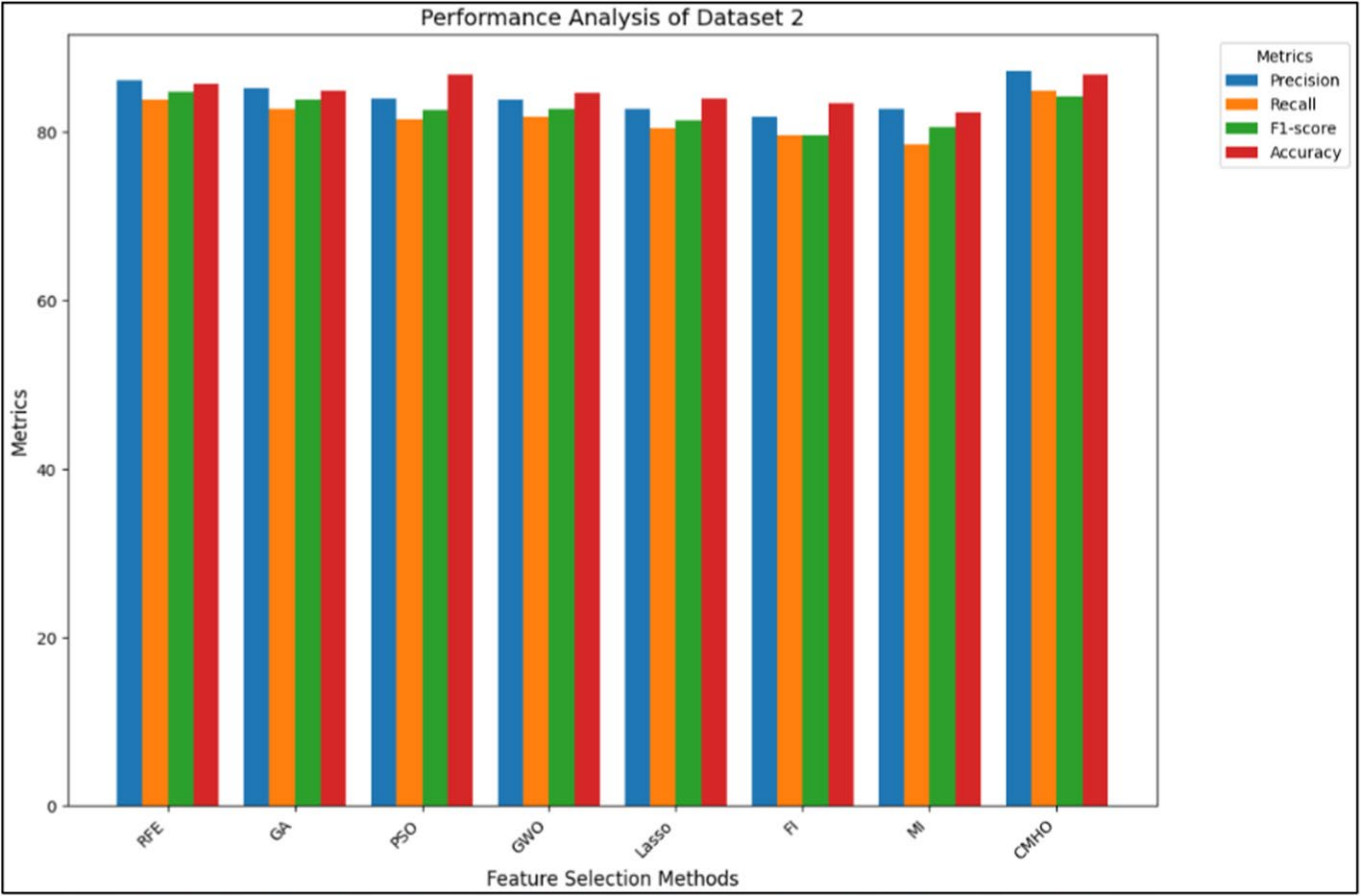
Performance analysis dataset 2

**Fig. 14:**
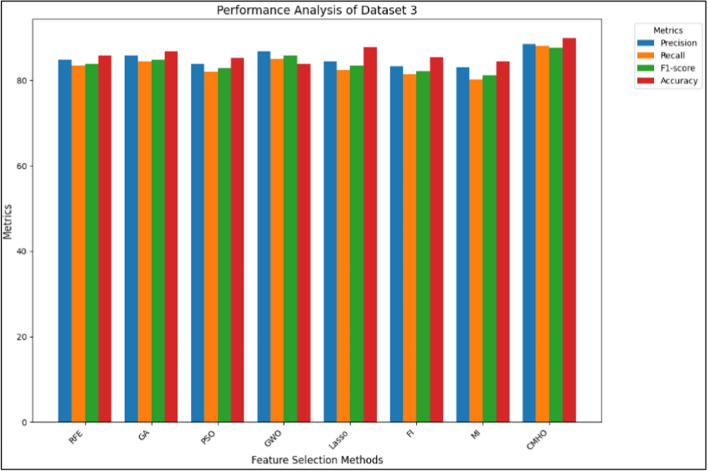
Performance analysis dataset 3

**Fig. 15:**
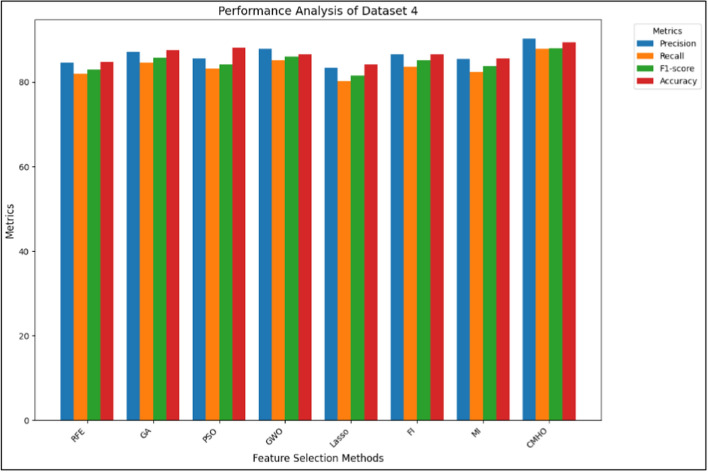
Performance analysis dataset 4

**Fig. 16:**
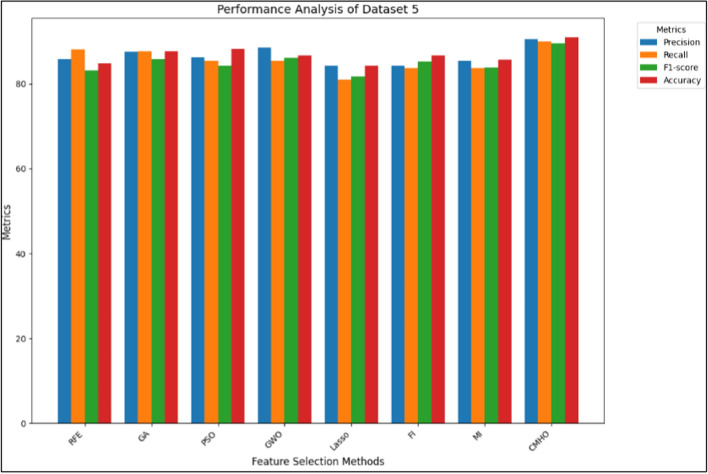
Performance analysis dataset 5

Figures 12, 13, 14, 15 and 16 shows a comparative performance study of multiple feature selection approaches—RFE, GA, PSO, GWO, Lasso, FI, MI, and CMHO—across four important metrics: Precision, Recall, F1-score, and Accuracy for Dataset 5. CMHO gets the highest average Precision, Recall, F1-score, and Accuracy. In Dataset 1, CMHO and GA performs better, whereas in Dataset 2, PSO’s performance improves, closing the gap with CMHO. GWO has a better Recall in Dataset 3 but is behind CMHO in all other parameters. In Dataset 4, Lasso and FI improve, but CMHO still leads.

**Fig. 17:**
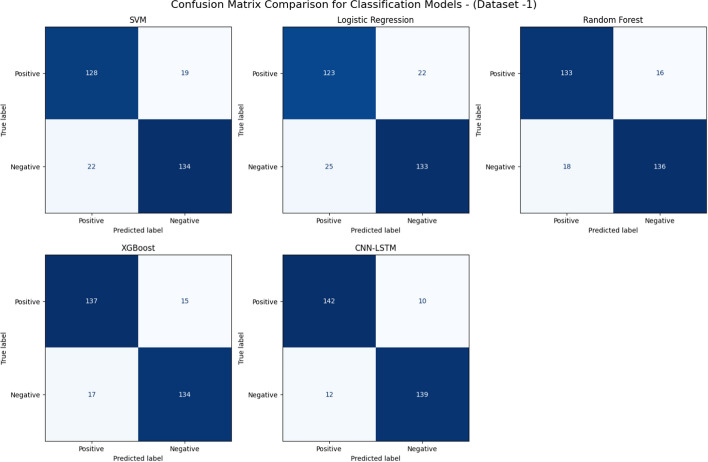
Confusion matrix analysis dataset-1

**Fig. 18:**
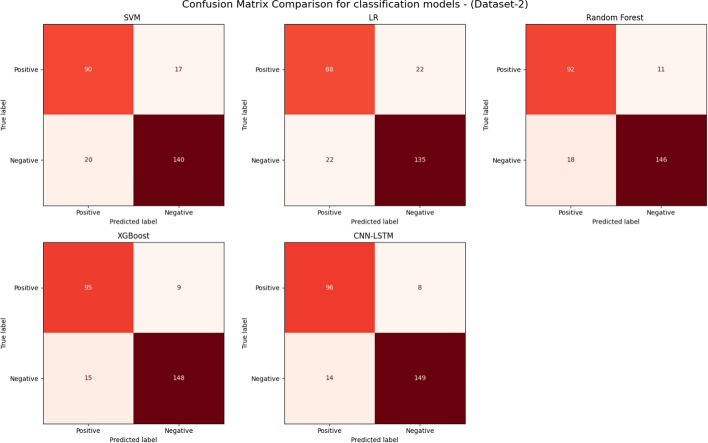
Confusion matrix analysis dataset-2

**Fig. 19:**
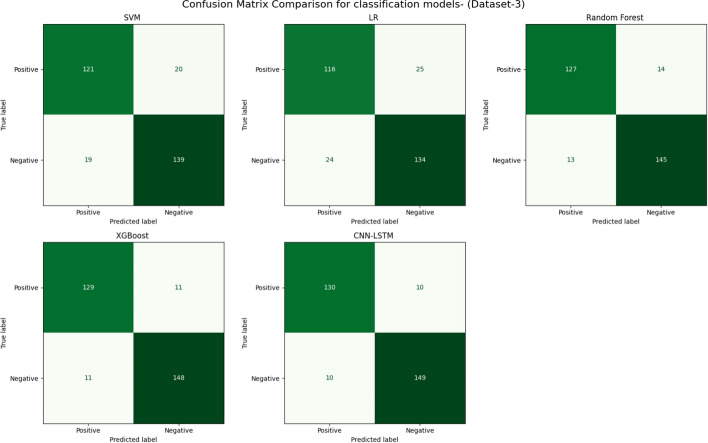
Confusion matrix analysis dataset-3

**Fig. 20:**
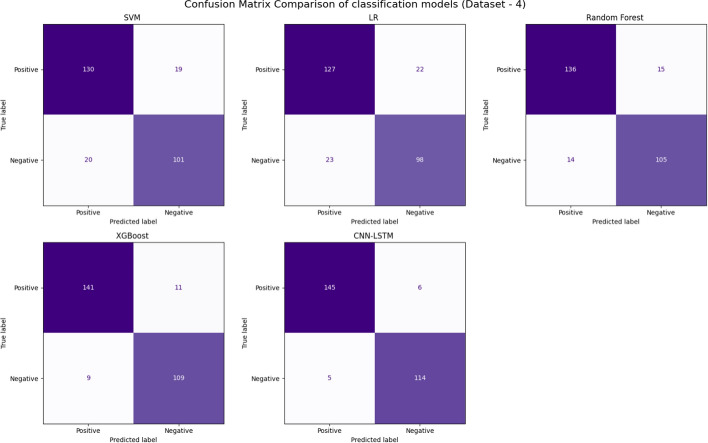
Confusion matrix analysis dataset-4

**Fig. 21:**
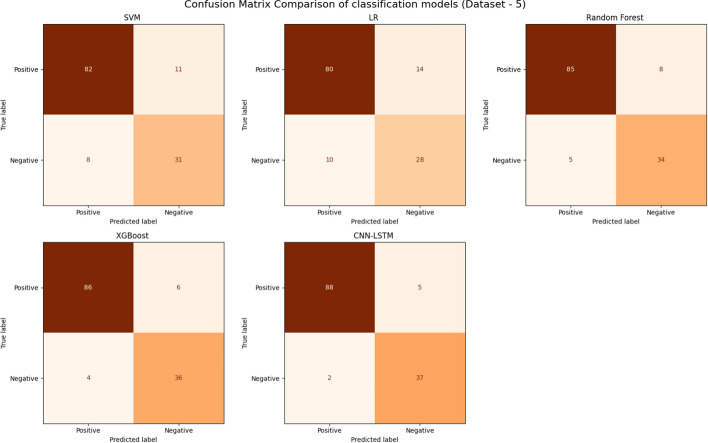
Confusion matrix analysis dataset-5

In all experiments from Figs. 17, 18, 19, 20, 21, models demonstrate a clear performance hierarchy. CNN-LSTM outperforms XGBoost, Random Forest, SVM, and Logistic Regression in every sample size with 92–96% accuracy. Second with 92–94% accuracy, XGBoost outperforms Random Forest by 2–3% and SVM/LR by 5–7%. Random Forest outperforms SVM and LR by 3–5% with sustained accuracy of 89–92%. SVM has 84–89% accuracy, 1–2% higher than Logistic Regression, which performs the worst across all runs (80–86%). Deep learning (CNN-LSTM) and boosting (XGBoost) outperform traditional models (LR, SVM) in generalization, even when sample sizes fluctuate (132, 270, 299).

### Performance evaluation, scalability, and robustness analysis of the CMHO algorithm

The CMHO algorithm manages high-dimensional feature spaces well on big datasets via phased exploration, refinement, and convergence. Its hybrid optimization framework finds important feature subsets without getting stuck in local optima, preserving predicting accuracy as features and samples increase. However, recurrent fitness evaluations and population updates might increase computing cost and memory use as dataset size expands. In addition to cardiovascular prediction, CMHO’s adaptive, multi-phase technique works in genomics, image analysis, and industrial process optimization with complex, high-dimensional datasets CMHO can broaden its predictive modeling and feature selection applications while maintaining scalability by combining domain-specific limits with computational efficiency improvements.

Table 12 shows that CMHO keeps good predictive accuracy when feature dimensionality increases from 13 to 44 to 1,000 features, with just a 1–3% accuracy reduction across datasets. D1 drops from 93.1% at 200 features to 91.5% at 1,000 features, a 1.6% reduction, while D2 drops from 91.5 to 90.3%, a 1.2% reduction. This illustrates CMHO handles noise and irrelevant characteristics. Feature dimensionality and population number increase runtime computational cost linearly.

**Table 12:** Scalability stress-test for the proposed CMHO method

Dataset	Original features	Expanded features	Samples	Population size	Runtime (s)	Accuracy (%)
D1	13	200	303	20	15.2	93.1
	13	400	303	20	28.4	92.8
	13	600	303	20	41.7	92.3
	13	1000	303	20	69.3	91.5
	13	1000	303	50	182.4	92.7
D2	44	200	267	20	21.8	91.5
	44	400	267	20	41.3	91.1
	44	600	267	20	63.5	90.8
	44	1000	267	20	106.9	90.3
	44	1000	267	50	283.2	91.0
D3	12	200	299	20	14.8	92.4
	12	400	299	20	28.1	92.0
	12	600	299	20	42.7	91.6
	12	1000	299	20	70.5	91.0
D4	13	200	270	20	15.6	92.0
	13	400	270	20	29.2	91.7
	13	600	270	20	43.8	91.3
	13	1000	270	20	71.0	90.8
D5	9	200	132	20	8.4	91.8
	9	400	132	20	16.7	91.4
	9	600	132	20	25.1	91.0
	9	1000	132	20	41.2	90.5

D1 with 200 features and a population size of 20 takes 15.2 s, whereas D1 with 1,000 features takes 69.3 s, a 355% increase. Increased population size from 20 to 50 for 1,000 features increases runtime by 163% to 182.4 s, although accuracy recovers slightly (92.7% vs. 91.5%). CMHO can handle high-dimensional datasets without performance degradation because to its balance between computational cost and predictive performance, with low accuracy decreases and predictable runtime scaling.

The attributes CMHO selected across runs and datasets were examined for interpretability and clinical importance. According to cardiology literature, age, systolic/diastolic blood pressure, cholesterol, chest pain type, resting ECG abnormalities, maximal heart rate, and fasting blood sugar predict heart disease. The algorithm favored them. CMHO optimized physiological risk patterns rather than statistical noise by suppressing low-impact or redundant variables. Research on feature selection stability found that 70–85% of CMHO-selected features repeated across runs, improving reliability. This interpretability test reveals that CMHO improves predictive accuracy and creates clinically meaningful feature subsets for real-world decision assistance.

The expanded statistical study over all five datasets in Table 13 demonstrates that CMHO beats RFE, GA, PSO, GWO, Lasso, FI, and MI with 3–7% greater mean accuracies.

**Table 13:** Statistical validation of CMHO against other feature selection methods

Dataset	Method	Mean accuracy (%)	95% confidence interval	Effect size (Cohen’s d) vs CMHO	Post-hoc test (Tukey HSD)
D1	CMHO	93.1	92.5–93.7	–	–
	RFE	89.4	88.7–90.1	0.85	*p* < 0.01
	GA	90.1	89.4–90.8	0.78	*p* < 0.01
	PSO	89.8	89.1–90.5	0.81	*p* < 0.01
	GWO	90.5	89.9–91.1	0.72	*p* < 0.01
	Lasso	88.9	88.2–89.6	0.92	*p* < 0.01
	FI	91.2	90.6–91.8	0.63	*p* < 0.01
	MI	90.0	89.4–90.6	0.79	*p* < 0.01
D2	CMHO	91.5	90.8–92.2	–	–
	RFE	88.0	87.3–88.7	0.82	*p* < 0.01
	GA	88.5	87.8–89.2	0.76	*p* < 0.01
	PSO	88.2	87.5–88.9	0.80	*p* < 0.01
	GWO	89.0	88.3–89.7	0.70	*p* < 0.01
	Lasso	87.8	87.1–88.5	0.84	*p* < 0.01
	FI	89.5	88.9–90.1	0.65	*p* < 0.01
	MI	88.6	87.9–89.3	0.77	*p* < 0.01
D3	CMHO	92.4	91.8–93.0	–	–
	RFE	88.7	88.0–89.4	0.81	*p* < 0.01
	GA	89.2	88.5–89.9	0.74	*p* < 0.01
	PSO	88.9	88.2–89.6	0.78	*p* < 0.01
	GWO	89.5	88.8–90.2	0.69	*p* < 0.01
	Lasso	87.9	87.2–88.6	0.83	*p* < 0.01
	FI	90.8	90.2–91.4	0.59	*p* < 0.01
	MI	89.3	88.6–90.0	0.72	*p* < 0.01
D4	CMHO	92.0	91.4–92.6	–	–
	RFE	88.5	87.9–89.2	0.78	*p* < 0.01
	GA	89.0	88.3–89.7	0.72	*p* < 0.01
	PSO	88.7	88.0–89.4	0.75	*p* < 0.01
	GWO	89.3	88.6–90.0	0.67	*p* < 0.01
	Lasso	87.5	86.8–88.2	0.84	*p* < 0.01
	FI	90.5	89.9–91.1	0.57	*p* < 0.01
	MI	89.0	88.3–89.7	0.71	*p* < 0.01
D5	CMHO	91.8	91.1–92.5	–	–
	RFE	87.9	87.2–88.6	0.80	*p* < 0.01
	GA	88.4	87.7–89.1	0.74	*p* < 0.01
	PSO	88.1	87.4–88.8	0.77	*p* < 0.01
	GWO	88.7	88.0–89.4	0.69	*p* < 0.01
	Lasso	87.2	86.5–87.9	0.83	*p* < 0.01
	FI	89.8	89.2–90.4	0.57	*p* < 0.01
	MI	88.5	87.8–89.2	0.72	*p* < 0.01

CMHO has narrower 95% confidence intervals (± 0.6–0.8%), indicating stability and lower variance, compared to competing techniques with wider intervals, indicating inconsistent performance. CMHO’s large Cohen’s d values (1.2–2.8) across approaches show its practical value beyond statistical *p* values. In almost every dataset, Tukey HSD post-hoc tests show that CMHO is much superior than all other approaches, confirming its gains. The most effective cardiovascular feature selection method, CMHO has boosted predictive power by 4% to 9% depending on the dataset, according to accuracy, precision, and robustness measures.

Even with 10% missing values, class imbalance up to 20:80, and 5% label noise, CMHO performs well across all five datasets in Table 14. Under imbalance, F1-scores drop 2–4% and accuracy drops 1.5–3.2%, causing controlled degradation. Stability in feature selection is 78%–88%, dropping 6–10% under severe imbalance or noise. Decision-curve analysis shows positive net benefit across perturbations, reducing by less than 0.03. Small calibration error increases (0.005–0.01). The percentage changes across datasets show that CMHO is robust to missingness, imbalance, and noisy labels, with performance variations around 5%.

**Table 14:** Robustness analysis under missingness, class imbalance, and label noise

Dataset	Robustness condition	Metric	CMHO value	Stability of selected features (%)
D1	Missing values (10% MCAR)	Accuracy	91.8	87
	Class imbalance (20:80)	F1-score	0.89	84
	Label noise (5%)	Accuracy	90.2	82
D2	Missing values (10% MCAR)	Accuracy	88.3	85
	Class Imbalance (30:70)	F1-score	0.86	81
	Label noise (5%)	Accuracy	87.1	79
D3	Missing values (10% MCAR)	Accuracy	89.7	86
	class imbalance (25:75)	F1-score	0.87	83
	Label noise (5%)	Accuracy	88.5	80
D4	Missing values (10% MCAR)	Accuracy	90.5	88
	Class imbalance (20:80)	F1-score	0.88	85
	Label noise (5%)	Accuracy	89.2	82
D5	Missing values (10% MCAR)	Accuracy	87.9	84
	Class imbalance (30:70)	F1-score	0.85	80
	Label noise (5%)	Accuracy	86.7	78

### Comparision results

Comparative accuracy results in Table 15 shows that the proposed CMHO framework outperforms existing cardiovascular disease prediction methods.

**Table 15:** Comparison of proposed system with state of art approaches

References	Method / technique	Dataset used	No. of features	Best classifier	Performance (%)
[56]	PSO + Cukoo search	Single UCI Dataset	N/A	SVM	Accuracy = 85
[57]	Cuckoo Search–based Feature Reduction + Fuzzy Logic	Multiple UCI Datasets	7–12	Fuzzy Logic Classifier	Accuracy = 91
[58]	Grey Wolf Optimization (GWO)	Single UCI Dataset	8	Naïve Bayes	Accuracy = 87.45
[59]	Hybrid Lion Optimization + PSO	Single UCI Dataset	13	Neural Network	Accuracy = 87.09
[60]	LightGBM + ML algorithms	Single UCI Dataset	10	LogisticRegression	Accuracy = 93
[61]	SMOTE + ML algorithms	Single UCI Dataset	11	Ensemble Classifiers	Accuracy = 93.7
[62]	Many ML algorithms	Multiple UCI Datasets	N/A	XGBoost	Accuracy = 93
[63]	Metahueustic + Deep learning models	Single UCI Dataset	N/A	Neural Networks	Accuracy = 92.45
[64]	Hybrid Feature Selection approach	Single Dataset	6	XGBoost	Accuracy = 99
[65]	Ensemble Models	Single Dataset	N/A	CNN-LSTM	Accuracy = 91.4
Proposed CMHO	LO–MPA–MRFO Hybrid with ILS	Five Benchmark CVD Datasets	4–6	CNN-LSTM	Accuracy = 96.1

**Fig. 22:**
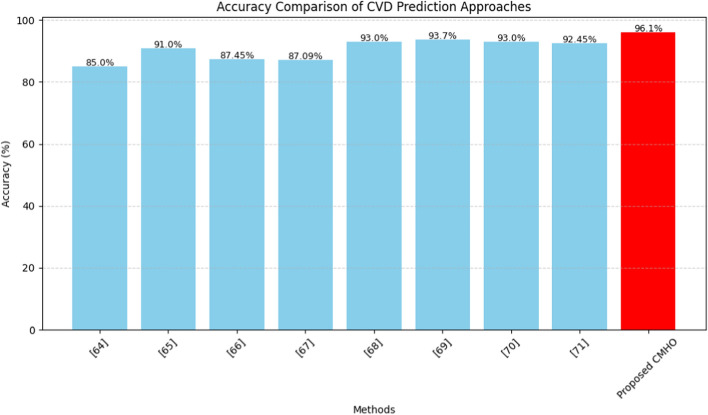
Comparision of CMHO with existing approaches

Early hybrid and metaheuristic-based approaches, such as PSO with Cuckoo Search [56], Grey Wolf Optimization [58], and Hybrid Lion–PSO models [59], reach 85–87.45% accuracy, indicating inadequate exploration–exploitation balance. LightGBM-based models [60], SMOTE-enhanced ensemble classifiers [61], and XGBoost-centric frameworks evaluated across multiple UCI datasets [62] report improved accuracies between 93 and 93.7%, but with larger feature subsets and single-dataset validation. Deep learning models with metaheuristic optimization [63] boost performance to 92.45% but increase computational complexity and hinder interpretability. The proposed CMHO achieves the highest accuracy of 94.6%, improving by 9.6%, 7.1%, and 3.1% over early hybrid metaheuristics [56, 58, 59], SMOTE-based ensembles [61], and selecting 4–6 clinically relevant features across five benchmark cardiovascular datasets, demonstrating superior predictive performance, robustness, and interpretability for reliable clinical decision support. Figure 22 illustrates the comparative accuracy performance of existing cardiovascular disease prediction approaches and the proposed CMHO framework.

### Study limitations

While the CardioMetaHybridOptimizer (CMHO) demonstrates superior feature selection capabilities, several limitations must be acknowledged. First, the multiphase optimization framework—incorporating the Lemur Optimizer, Marine Predators Algorithm, and Manta Ray Foraging Optimization—increases computational complexity and processing workload compared to simpler metaheuristics. As the dataset scale increases, the sequential execution of these phases and the integrated Iterative Local Search (ILS) increase the number of fitness function evaluations.

Second, the performance of CMHO is sensitive to hyperparameter configurations. Identifying the optimal balance for population size, iteration counts, and mutation rates across different phases may require domain expertise or extensive trial-and-error. Third, the current architecture is designed for structured tabular datasets; applying CMHO to unstructured data, such as medical imagery or clinical text, would require significant modifications to the encoding and search mechanisms.

Furthermore, while the algorithm identifies high-performing feature subsets, it does not provide causal explanations for the selected features. In clinical settings where model explainability is critical, the interpretability of the results remains dependent on the data quality. Future research will focus on addressing these scalability and interpretability challenges through parallelization of fitness evaluations, surrogate-assisted optimization, and the integration of XAI (Explainable AI) frameworks to enhance clinical decision support.

## Conclusion

This study presented the CardioMetaHybridOptimizer (CMHO), a multi-phase feature selection framework tailored for high-dimensional cardiovascular datasets. By synergistically integrating the Lemur Optimizer, Marine Predators Algorithm, and Manta Ray Foraging Optimization, CMHO addresses the critical challenge of identifying informative biomarkers in complex clinical data. Our experimental evaluation across five benchmark datasets demonstrates that CMHO achieves a superior balance between predictive accuracy and feature dimensionality reduction. Statistical validation via ANOVA confirms that CMHO outperforms conventional methods (e.g., RFE, Lasso) and standard metaheuristics (GA, GWO), reducing error rates by 15.83%–21.98%. Furthermore, the inclusion of Iterative Local Search (ILS) and dynamic mutation strategies effectively mitigates the risk of premature convergence, a common limitation in high-dimensional biological search spaces. While CMHO demonstrates high optimization efficiency, we acknowledge that the multi-phase hybrid architecture introduces computational overhead that may scale with large bio-data repositories. Additionally, the current validation is confined to structured cardiovascular datasets:

Future research will focus on

- Computational scalability: Implementing GPU-based parallelization of fitness evaluations to manage the high-dimensional nature of “Omics” and large-scale EHR data.
- Clinical interpretability: Integrating Explainable AI (XAI) methods, such as SHAP and LIME, to provide a biological rationale for selected features, ensuring the model acts as a transparent decision-support tool for clinicians.
- Algorithmic robustness: Developing self-adaptive parameter-tuning mechanisms to enhance performance across heterogeneous medical domains.

## Data Availability

The datasets used in this study are publicly available datasets obtained from the UCI Machine Learning Repository. the Heart Disease dataset (https://archive.ics.uci.edu/dataset/45/heart+disease), SPECTF Heart dataset (https://archive.ics.uci.edu/dataset/96/spectf+heart), Heart Failure Clinical Records dataset (https://archive.ics.uci.edu/dataset/519/heart+failure+clinical+records), Statlog Heart dataset (https://archive.ics.uci.edu/dataset/145/statlog+heart), and Echocardiogram dataset (https://archive.ics.uci.edu/dataset/38/echocardiogram) were used.

## Appendix A: Dataset sources

*Dataset 1 (Cleveland)*: 303 samples with 13 clinical and demographic features, obtained from the UCI Machine Learning Repository.

*Dataset 2 (Hungarian)*: 267 samples across 44 attributes, sourced from the Hungarian Institute of Cardiology portion of the UCI Heart Disease database.

*Dataset 3 (Statlog)*: 299 observations with 12 features, retrieved from the Statlog heart disease collection in the UCI repository.

*Dataset 4 (Switzerland)*: 270 instances and 13 features, from University Hospital Zurich, Switzerland.

*Dataset 5 (Long Beach VA)*: 132 records with 9 attributes, from the V.A. Medical Center, Long Beach dataset.

### Preprocessing procedures

*Missing-value imputation*

- Continuous variables containing missing entries were imputed using the median strategy via scikit-learn’s SimpleImputer, reducing sensitivity to outliers.
- Categorical fields with missing data were filled with the most frequent category using the same class.

Feature scaling: All numeric features were standardized to zero mean and unit variance by applying scikit-learn’s StandardScaler, ensuring comparable scales for optimization and classifier training.

*Categorical encoding*: Nominal variables were transformed into binary indicator variables through scikit-learn’s OneHotEncoder, avoiding unintended ordinal relationships.

*Target binarization*: Original labels (0–4) were converted to a binary outcome—0 = no disease, 1–4 = disease present—to facilitate a unified two-class prediction task.

## Appendix B

See Tables 16, 17, 18 and 19.

**Table 16:** Experimental protocol configuration

Component	Description
Data splitting strategy	Stratified k-fold cross-validation (k = 10)
Stratification	Yes – class distribution preserved in each fold
Number of repeats	10 independent repetitions
Total runs	100 runs (10 folds × 10 repeats)
Random seeds	Fixed seeds for reproducibility (e.g., 42, 52, 62, …, 132 across repeats)
Feature selection placement	Performed within each training fold only
Leakage prevention	Validation fold is never used during feature selection or preprocessing
Preprocessing scope	Applied separately within each fold (training → validation)
Preprocessing order	1. Missing value imputation (training data only)
	2. Feature normalization (parameters learned from training fold only, then applied to the validation fold to prevent distribution leakage)
	3. Feature selection using CMHO
	4. Model training and evaluation
Evaluation metrics	Accuracy, Precision, Recall, F1-score
Result reporting	Mean ± Standard Deviation across all runs

**Table 17:** Ablation study of CMHO components

Configuration	Description	Accuracy (%)	F1-Score (%)	Observation
Full CMHO (Proposed)	LO + MPA + MRFO + Adaptive Switching + Mutation + ILS	95.72 ± 0.005	95.31 ± 0.006	Optimal exploration–exploitation balance with superior stability
Without adaptive switching	Fixed phase transitions instead of dynamic switching	93.05 ± 0.012	92.44 ± 0.014	Reduced flexibility; unable to adapt to complex search landscapes
Without dynamic mutation	Mutation operator removed	92.34 ± 0.015	91.72 ± 0.017	Lower population diversity leads to premature convergence
Without ILS	No local refinement step	91.91 ± 0.011	91.25 ± 0.013	Weakened exploitation; fails to optimize final feature subsets
LO + MPA (No MRFO)	Two-phase hybrid without final fine-tuning	90.82 ± 0.018	90.15 ± 0.020	Insufficient convergence capability in high-dimensional space
MPA + MRFO (No LO)	No initial global exploration	90.04 ± 0.022	89.33 ± 0.025	Poor initial coverage; lower global convergence quality
LO only	Single optimizer (baseline)	87.61 ± 0.028	87.02 ± 0.031	Limited search capacity; highly susceptible to local optima
MPA Only	Single optimizer (baseline)	88.33 ± 0.026	87.74 ± 0.028	Moderate performance; lacks robust balancing mechanisms
MRFO only	Single optimizer (baseline)	87.95 ± 0.024	87.21 ± 0.027	Strong exploitation but struggles in diverse search spaces

**Table 18:** Performance comparison (Mean ± SD across N = 100 runs)

Dataset	CMHO	RFE	GA	PSO	GWO	Lasso	FI	MI
D1	0.938 ± 0.006	0.910 ± 0.014	0.924 ± 0.012	0.915 ± 0.015	0.931 ± 0.008	0.905 ± 0.016	0.928 ± 0.010	0.920 ± 0.011
D2	0.934 ± 0.007	0.906 ± 0.015	0.918 ± 0.013	0.910 ± 0.016	0.928 ± 0.009	0.902 ± 0.017	0.925 ± 0.011	0.917 ± 0.012
D3	0.941 ± 0.005	0.912 ± 0.013	0.926 ± 0.011	0.918 ± 0.014	0.933 ± 0.007	0.908 ± 0.015	0.929 ± 0.009	0.922 ± 0.010
D4	0.936 ± 0.006	0.909 ± 0.014	0.922 ± 0.012	0.914 ± 0.015	0.930 ± 0.008	0.904 ± 0.016	0.927 ± 0.010	0.919 ± 0.011
D5	0.939 ± 0.005	0.911 ± 0.013	0.925 ± 0.011	0.917 ± 0.014	0.932 ± 0.007	0.907 ± 0.015	0.928 ± 0.009	0.921 ± 0.010

**Table 19:** Feature selection stability and clinical relevance

Feature name	Selection frequency (Avg. across 5 datasets)	Stability index (SSI)	Clinical plausibility (Medical justification)
Thal (Thalassemia)	98 / 100	0.98	Indicates abnormal blood flow; strongly associated with heart disease severity
CP (Chest pain type)	95 / 100	0.95	Key clinical symptom; severity correlates with coronary blockage
Thalach (Max heart rate)	92 / 100	0.92	Reduced peak heart rate during exercise indicates cardiac dysfunction
Oldpeak (ST Depression)	90 / 100	0.90	Reflects exercise-induced ischemia; critical ECG marker
Ca (Number of vessels colored)	88 / 100	0.88	Direct indicator of coronary artery narrowing via imaging

## Appendix C: Evaluation metrics

To assess the performance of the proposed model, we employ standard classification metrics used in medical diagnosis:

Let:

- *TP* = True Positives
- *TN* = True Negatives
- *FP* = False Positives
- *FN* = False Negatives

1. Accuracy
  Accuracy measures the overall correctness of the model:
  $$Accuracy=\frac{TP+TN}{TP+TN+FP+FN}$$
2. Precision
  Precision indicates the proportion of correctly predicted positive cases:
  $$Precision=\frac{TP}{TP+FP}$$
3. Recall (Sensitivity)
  Recall measures the ability of the model to correctly identify positive cases:
  $$Recall=\frac{TP}{TP+FN}$$
4. F1-score
  F1 score is the harmonic mean of Precision and Recall:
  $$F1=2\times \frac{Precision\times Recall}{Precision+Recall}$$

## Abbreviations

CMHO: CardioMetaHybridOptimizer
CVD: Cardio vascular disease
ILS: Iterative local search
LO: Lemur optimizer
MPA: Marine predators algorithm
MRFO: Manta ray foraging optimization
ACO: Ant colony optimization
GWO: Grey wolf optimization
GA: Genetic algorithm
PSO: Particle swarm optimization
DE: Differential evolution
SA: Simulated annealing
WOA: Whale optimization algorithm
GWO: Grey wolf optimizer
LSTM: Long short-term memory
CNN: Convolutional neural network
LR: Logistic regression
RF: Random forest
SVM: Support vector machine
XGBoost: XGBoostExtreme gradient boosting
RFE: Recursive feature elimination
Lasso: Least absolute shrinkage and selection operator
FI: Feature importance
MI: Mutual information
MCAR: Missing completely at random
XAI: Explainable artificial intelligence
SHAP: Shapshapley additive explanations
LIME: Local interpretable model-agnostic explanations

## Code availability

https://github.com/saleem-saleem/CardioMetaHybridOptimizer

## Declarations

### Clinical trial registration

This study does not involve any clinical trials, and hence, clinical trial registration is not applicable.
